# Identification and Characterization of Mitogen-Activated Protein Kinase (MAPK) Genes in Sunflower (*Helianthus annuus* L.)

**DOI:** 10.3390/plants8020028

**Published:** 2019-01-22

**Authors:** Surendra Neupane, Sarah E. Schweitzer, Achal Neupane, Ethan J. Andersen, Anne Fennell, Ruanbao Zhou, Madhav P. Nepal

**Affiliations:** 1 Department of Biology and Microbiology, South Dakota State University, Brookings, SD 57007, USA; surendra.neupane@sdstate.edu (S.N.); sarah.schweitzer@jacks.sdstate.edu (S.E.S.); achal.neupane@sdstate.edu (A.N.); ethan.andersen@sdstate.edu (E.J.A.); ruanbao.zhou@sdstate.edu (R.Z.); 2 Department of Agronomy, Horticulture and Plant Science, South Dakota State University, Brookings, SD 57007, USA; anne.fennell@sdstate.edu

**Keywords:** Abiotic stress, cellular signaling, protein kinase, MAPK cascade, MAPK nomenclature, sunflower, RNA-seq

## Abstract

Mitogen-Activated Protein Kinase (MAPK) genes encode proteins that regulate biotic and abiotic stresses in plants through signaling cascades comprised of three major subfamilies: MAP Kinase (MPK), MAPK Kinase (MKK), and MAPKK Kinase (MKKK). The main objectives of this research were to conduct genome-wide identification of MAPK genes in *Helianthus annuus* and examine functional divergence of these genes in relation to those in nine other plant species (*Amborella trichopoda*, *Aquilegia coerulea*, *Arabidopsis thaliana*, *Daucus carota*, *Glycine max*, *Oryza sativa*, *Solanum lycopersicum*, *Sphagnum fallax*, and *Vitis vinifera*), representing diverse taxonomic groups of the Plant Kingdom. A Hidden Markov Model (HMM) profile of the MAPK genes utilized reference sequences from *A. thaliana* and *G. max*, yielding a total of 96 MPKs and 37 MKKs in the genomes of *A. trichopoda*, *A. coerulea*, *C. reinhardtii*, *D. carota*, *H. annuus*, *S. lycopersicum*, and *S. fallax*. Among them, 28 MPKs and eight MKKs were confirmed in *H. annuus*. Phylogenetic analyses revealed four clades within each subfamily. Transcriptomic analyses showed that at least 19 HaMPK and seven HaMKK genes were induced in response to salicylic acid (SA), sodium chloride (NaCl), and polyethylene glycol (Peg) in leaves and roots. Of the seven published sunflower microRNAs, five microRNA families are involved in targeting eight MPKs. Additionally, we discussed the need for using MAP Kinase nomenclature guidelines across plant species. Our identification and characterization of MAP Kinase genes would have implications in sunflower crop improvement, and in advancing our knowledge of the diversity and evolution of MAPK genes in the Plant Kingdom.

## 1. Introduction

Plant responses to abiotic and biotic stresses involve protein kinases that are crucial to signal transduction pathways [1]. The protein kinases are involved in a phosphorylation of Serine/Threonine and Tyrosine sidechains of proteins [2]. Among these protein kinases, Mitogen-Activated Protein Kinase (MAPK) cascade genes are key components of signal transduction pathways in animals, plants, and fungi [3] that help transduce extracellular signals to intracellular responses [4]. Discovered in 1986, the MAPK gene family was originally found in animal cells as a microtubule-associated protein kinase [5]. The first reports of plant MAPK gene family in 1993, identified MsERK1 in alfalfa [6] and D5 kinase in pea [7]. MsERK1 is believed to play a role as an inducer of mitosis in root nodules during symbiosis by *Rhizobium* and D5 kinase as a cell cycle regulator in pea [6,7]. In addition to such roles in cell proliferation and cell differentiation, MAPK genes are involved in regulating various biotic (e.g., bacteria, fungi, viruses) and abiotic (e.g., light, drought, UV, salinity, pH, cold) stress responses [8].

Stress signals trigger the MAPK cascade, which is composed of reversibly phosphorylated kinases such as MAP Kinase (MAPK, MPK), MAPK Kinase (MAP2K, MAPKK, MKK), and MAPKK Kinase (MAP3K, MAPKKK, MKKK) [9,10]. The MKKKs constitute a relatively larger gene family, constituting three sub-groups of genes: the MEKKs, Rafs, and ZIKs [11]. Each of these proteins in the cascade is activated through the recognition and phosphorylation of a specific serine/threonine amino acid motif [12]. An external or internal stimulus triggers the first step, the activation of an MKKK member, through receptor-mediated phosphorylation or intermediate bridging factors or interlinking MKKKs [10]. The phosphorylated MKKK member induces the activation of MKK through the phosphorylation of two serine or threonine amino acid residues in the conserved motif S/TxxxxxS/T [10]. The activated MKKs, which are dual-specificity kinases, in turn, trigger the phosphorylation of MPKs at the Thr-Asp/Glu-Tyr [T(D/E)Y] motif located in the activation loop (T-loop) between kinase subdomains VII and VIII [3,10,13]. Apart from T(D/E)Y motif in many plant species, some other variants such as T(Q/V/S)Y, T(/Q/R)M, MEY, TEC in the activation loop have also been reported [1]. The MPK members phosphorylate a variety of substrates, including transcription factors, protein kinases, and cytoskeleton proteins [10,14]. The activation of the MAPK cascade genes induces the translocation from the cytoplasm to the nucleus [15], further enacting the specific cellular response to the external stimuli through gene activation and inactivation. The detailed illustration of the MAP Kinase signaling pathway in response to diverse abiotic and biotic stresses in plants is represented in Figure S1 adapted from various studies [16,17,18,19,20,21,22,23,24].

The advent of sequencing technologies and rapid progress in bioinformatics tools has assisted the sequencing of the plant genomes at a faster pace. Genome-wide identification of MPKs and MKKs has been documented in various plant species, including both model and crop species [14,25,26,27,28,29,30,31,32,33,34,35,36,37,38,39]. Previous identification and characterization of MAP Kinase cascade proteins in rice, *Arabidopsis*, and other plants [4,39,40] provide a wealth of information for comparative analyses of these proteins in species that have yet to be studied. The availability of the complete genome sequences from each of the major plant groups such as Asterids (*Daucus carota* [41], *Helianthus annuus* [42], *Solanum lycopersicum* [43]), Amborellales (*Amborella trichopoda* [44]), Ranunculales (*Aquilegia coerulea* [45]), Bryophyte (*Sphagnum fallax* [46]), and Algae (*Chlamydomonas reinhardtii* [47]) allowed us to identify the MPK and MKK genes of these species and assess phylogenetic relationships. Domesticated sunflower is the fourth most important oilseed crop in the world (http://www.fao.org/) and can adapt to diverse environmental conditions such as drought and maintain the stable yields [48]. Thus, the MAPK gene family might play an important role in helping sunflower adapt and survive in different environmental conditions. This research was carried out with two major objectives: (a) detailed identification and functional characterization of MPK and MKK genes in *H. annuus*; and (b) assess phylogenetic relationships of MPK and MKK genes of *H. annuus* with that of *A. coerulea*, *A. trichopoda*, *C. reinhardtii*, *D. carota*, *S. fallax*, and *S. lycopersicum* and including the homologs from relatively better-studied plant species from Rosids (*A. thaliana*, *G. max*, and *V. vinifera*) and a monocot (*O. sativa*). Findings from this study might support further efforts in crop improvement focused on the development of cultivars that maintain yield when challenged by biotic and abiotic stresses as well as understand the evolution pattern of MAPK gene family in sunflower and other plant species.

## 2. Materials and Methods

### 2.1. Retrieval and Identification of Putative MAP Kinase Cascade Genes

**Figure 1:**
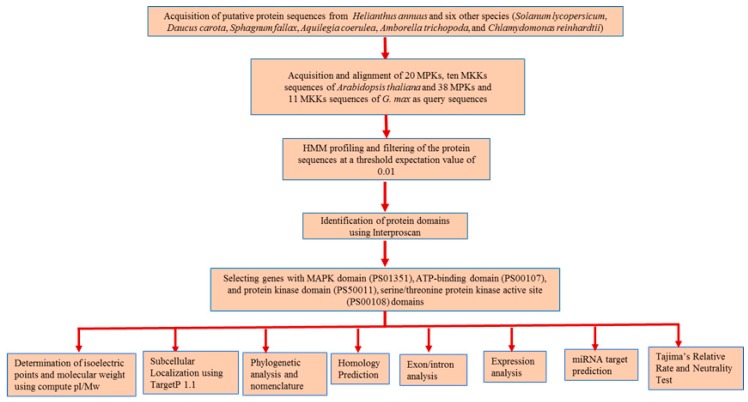
Schematic representation of *in silico* approaches used in the identification of MPK and MKK genes in seven plant species and their downstream analyses.

Genome-wide identification of MPK and MKK cascade genes was performed using protein sequences of *A. coerulea* (v 3.1), *A. trichopoda* (v 1.0), *C. reinhardtii* (v 5.5), *D. carota* (v 2.0), *H. annuus* (r 1.2), *S. fallax* (v 0.5), and *S. lycopersicum* (iTAG2.4) obtained from the Phytozome database [45]. Sunflower protein sequences from INRA inbred genotype XRQ whose genome is 3.6 gigabases and encodes 52,243 proteins distributed over 17 chromosomes [42] were analyzed in the present study. The 20 MPK and ten MKK sequences of *A. thaliana* [25] along with 38 MPK and 11 MKK sequences of *G. max* [26] were used as reference sequences for the identification of MPK and MKK proteins. The multiple sequence alignment of these reference sequences was employed in HMM profiling using the program HMMER (version 3.1b2) [49] at a threshold e-value of 0.01. MPK and MKK genes were further identified using InterProScan (version 5.27) [50], Pfam ID [51], and PROSITE ID (http://prosite.expasy.org/). The proteins with PfamID of MAPK domain (PS01351), ATP-binding domain (PS00107), protein kinase domain (PS50011), and serine/threonine protein kinase active site (PS00108) were used for identification of corresponding MPK and MKK proteins (Figure 1). Multiple expectation maximization for motif elicitation (MEME) [52] and multiple sequence alignment analysis was performed to confirm the presence of the following signature motifs: (a) the phosphate binding P-loop, GxGxxG [1], where ATP binds in protein kinases; (b) the catalytic C-loop, D(L/I/V)K, found within the S/T PK active site signature; and (c) the activation- or T-loop, T(D/E)Y in MPK and GTxxYMSPER in MKK proteins. The following parameters for MEME were employed: maxsize: 100,000; mod: zoops; nmotifs: 10; minw: 6; and maxw: 25. Furthermore, MKK genes were identified using BLAST [53], with an e-value cutoff of 0.01, in which *A. thaliana* MKK sequences were used as a query, and the top ten hits for each *A. thaliana* MKK query sequence were employed for MKK gene identification. The protein theoretical molecular weight and isoelectric point were predicted using compute pI/Mw tool available in ExPASy (http://au.expasy.org/tools). Subcellular localization of the putative MPK and MKK genes of sunflower were analyzed using TargetP 1.1 [54].

### 2.2. Phylogenetic Tree Construction and Homology Assessment

The multiple sequence alignment of identified MPK and MKK proteins of *H. annuus* and other species used in this study was performed using CLUSTALW [55] and MUSCLE [56] in Geneious [57] and subjected to phylogenetic analysis employing the maximum likelihood (ML; with 100 replicates) using MEGA (version 7.0.14) [58]. The phylogenetic analyses employed an evolutionary model ‘Jones-Taylor-Thornton with gamma distribution and invariant sites (JTT+G+I)’, the best evolutionary model resulted from the ModelTest analysis using MEGA7. The trees using MPK and MKK sequences were rooted with corresponding human MAPK proteins [HsMAPK1 (GenBank: NP_002736.3) and HsMAPKK1 (GenBank: AAI37460.1), respectively] as an outgroup. Timetree was constructed using the Reltime method [59] from MEGA7 to study the evolutionary divergence of MKK3 proteins belonging to all species under study. The following criteria were used for the construction of Timetree: constraints used: 3 [Divergence time: *O. sativa* and *A. trichopoda* (168–194 MYA), *G. max* and *H. annuus* (110–124 MYA), and *V. vinifera* and *A. thaliana* (105–115 MYA), obtained from http://www.timetree.org/ [60]]; variance estimation method: analytical; statistical method: maximum likelihood; substitution model: JTT; rates among sites: 5 categories (+*G*, parameter = 0.6307); rate variation model allowed: ([+I], 0.00% sites); amino acids involved: 11; and total positions: 574 positions. Homology to MPKs and MKKs of other plants was assessed using the BLASTp top-hit approach (https://blast.ncbi.nlm.nih.gov/Blast.cgi) with non-redundant protein sequences (nr) database.

### 2.3. Chromosomal Locations and Gene Structure

All 17 chromosome sequences of *H. annuus* accessed from the Phytozome database were uploaded into the program Geneious [57]. The chromosome locations of MPK and MKK genes of sunflower were visualized using annotation file in Generic Feature Format (GFF) obtained from the annotation database of Phytozome. The exon-intron distribution pattern was obtained by the Gene Structure Display Server [61].

### 2.4. Nomenclature of MPKs and MKKs

Nomenclature of sunflower MPKs and MKKs was carried out using MAPK gene nomenclature guidelines [3,4]. The nomenclature uses the following format: (a) the first letter (upper case) of the genus name followed by two to three letters of species (lower case) was used; (b) a number was provided based on the homology to the *Arabidopsis* MAPK cascade genes; and (c) the number was followed by a hyphen and a number if paralogs were present. Such guidelines for nomenclature of MPKs and MKKs have been employed in many studies [1,4,26,27,33,34,35,36,62,63,64,65]. In this study, we renamed GSVIVT01005924001 (VvMPK2) and GSVIVT0102277001 (VvMPK10), identified by Cakir and Kılıçkaya 2015 [37], as VvMPK22 and VvMPK23, respectively, which were not identified in a study by Mohanta et al. 2015 [1].

### 2.5. Expression Analysis and miRNA Prediction of Sunflower MPKs and MKKs

The expression pattern of sunflower MPKs and MKKs was investigated using data accessed from NCBI SRA SRP092742 [SRR4996815 (polyethylene glycol or peg)-treated pooled root samples), SRR4996819 (NaCl-treated pooled root samples), SRR4996823 (Peg-treated pooled leaf samples), SRR4996828 (pooled control root samples), SRR4996834 (NaCl-treated pooled leaf samples), SRR4996836 (pooled control leaf samples), SRR4996839 (salicylic acid-treated pooled leaf samples), and SRR4996847 (salicylic acid-treated pooled root samples)]. These data are the result of the application of one hormone treatment (0.05 µM SA), two abiotic stresses [Peg 6000 (100g/l), which creates osmotic stress, and NaCl (100mM) for salt stress], and control [dimethyl sulfoxide (DMSO) only] collected from root and leaf samples. The detailed experiment is described in Badouin et al. 2017 [42]. Briefly, roots and first leaves were collected after six hours of treatment (SA, Peg, NaCl, and DMSO), and applied to two-week-old sunflower seedlings (INRA inbred genotype XRQ) grown in a hydroponic system. The collection was repeated three times and was pooled after separate RNA extractions in equimolar concentration. RNA sequencing of root and leaf samples was performed as non-oriented pair end libraries (2*76bp for roots and 2*100 for leaves). The quality control of these reads was accessed by running the FastQC program (version 0.11.3) [66], and trimming was done using Btrim64 (version 0.2.0) [67] to remove low-quality bases (QC value > 20; 5-bp window size). High-quality pair-end reads were mapped against the coding sequences of *H. annuus* (*Hannuus: Hannuus_494_r1.2.transcript.fa.gz*) obtained from the Phytozome database using the Salmon (version 0.9.1) [68] in Bioconda [69]. The codes that were used for data processing are available as Supplementary File S1. The obtained transcript estimated quantification reads for each treatment were compared with their respective reads from the controls to calculate the log_2_Fold Change (log_2_FC) and visualized using integrated Differential Expression and Pathway analysis (iDEP 0.81 R/Bioconductor packages; http://bioinformatics.sdstate.edu/idep/) [70]. The heatmap was generated using the following criteria: distance–correlation, linkage–average and cut-off Z score–4 to study the hierarchical clustering and expression pattern of MPK and MKK genes in different tissues under different treatments. *k*-means clustering was done using the standardization normalization technique. For identifying the potential miRNA targeting sites, the nucleotide sequences of the identified sunflower MPKs and MKKs were subjected to a plant small RNA (psRNATarget) target analysis server [71] against seven published *H. annuus* microRNAs, selecting Schema V2 (2017 release) as a scoring option.

### 2.6. Tajima’s Relative Rate and Neutrality Test

Tajima’s relative rate test [72] was conducted to study the statistical significance of variations in molecular evolution in a different group of plants. The same MEGA files used in phylogenetic tree construction were used in the program MEGA7. In this test, three random sequences of either MPKs or MKKs of different plant species were selected, considering one of the sequences as the outgroup, and the χ^2^ test statistic was applied. A *p*-value of less than 0.05 was used to reject the null hypothesis of equal rates of evolution between selected sequences of different plant groups. All positions containing gaps and missing data were eliminated. Tajima’s test of neutrality [73] was performed to understand and distinguish the evolutionary pattern of randomly evolved MPKs or MKKs with non-randomly evolving MPKs or MKKs. During the neutrality test, all positions with less than 95% site coverage were eliminated. Therefore, fewer than 5% alignment gaps, missing data, and ambiguous bases were allowed at any position. The groupings of A, B, and C represent the statistical groups, which should not be confused with MPK or MKK clades.

## 3. Results

### 3.1. The Diversity of MPK and MKK Genes in Sunflower Relative to Other Species

After a careful examination of the signature motifs of the 2,419 sequences resulting from the HMM profiling using reference sequences of *A. thaliana* and *G. max* against 52,243 protein sequences of sunflower, we identified 28 MPKs (filtered from 244 possible MPKs) and eight MKKs (filtered from 100 possible MKKs) (Table 1 and Table 2). We also used protein sequences of *A. coerulea*, *A. trichopoda*, *C. reinhardtii*, *D. carota*, *S. fallax*, and *S. lycopersicum* and identified their MPKs and MKKs, which are shown in Tables S1 and S2. The protein sequences identified, including reference sequences used in this study and their identity in percentage, are presented in Supplementary File S2. The abundance of MPK and MKK genes in the genomes of *A. coerulea* (306.5 Mb), *A. trichopoda* (706 Mb), *C. reinhardtii* (111 Mb), *D. carota* (421 Mb), *H. annuus* (3600 Mb), *S. lycopersicum* (900 Mb), and *S. fallax* (395 Mb) shares no apparent correlation with genome size (Table 1).

### 3.2. Gene Location, Subcellular Localization and Structural Variation of MPKs and MKKs in H. annuus

**Figure 2:**
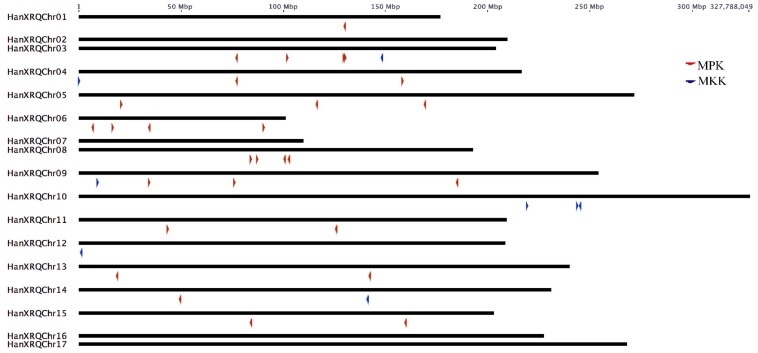
Chromosomal distribution of MPK and MKK genes in sunflower (*n* = 17). Color-coded arrows represent MAP Kinase gene types and their orientation on the chromosome indicated by the black line.

The MPK and MKK genes were distributed on all chromosomes of sunflower, with the highest of five genes in chromosome 3. The MPK genes were absent in chromosomes 2, 7, 10, 12, 10, 16, and 17; whereas, MKK genes were absent in chromosomes 1, 2, 5, 6, 7, 8, 11, 13, 15, 16, and 17. Both MPK and MKK genes are completely absent in chromosomes 2, 7, 16, and 17. One HaMPK gene each was found in chromosome 1 and 14; two HaMPKs each in chromosome 4, 11, 13, and 15; three HaMPKs each in chromosome 5 and 9, and four HaMPKs each in chromosome 3, 6 and 8 (Figure 2). Eight paralog pairs HaMPK3-1/3-2, HaMPK6-1/6-2, HaMPK9-1/9-2, HaMPK11-1/11-2, HaMPK13-1/13-2, HaMPK 16-1/16-2, HaMPK19-1/19-2, and HaMPK23-2/23-4 were located on different chromosomes. Only one paralog pair (HaMPK23-1/23-3) was present in the same chromosome (i.e., chromosome 3). Likewise, only one MKK gene was present in chromosomes 3, 4, 9, 12, and 14, while three MKKs were present in chromosome 10. The only paralog pair, HaMKK6-1/6-2 was present in different chromosomes. TargetP analysis showed that the proteins encoded by three MPKs (HaMPK11-1/11-2 and HaMPK4) and two MKKs (HaMKK9 and HaMKK3) were predicted to localize in mitochondria, two MKKs (HaMKK4 and HaMKK5) in the chloroplast, and the rest in subcellular locations other than mitochondria or the chloroplast (Table 2). Regarding the structural variation due to exons and introns, the number of exons in MPKs ranged from two (HaMPK4) to 18 (HaMPK22, HaMPK23-4/23-2) with an average of 8.9 exons per gene (Table 2, Figure S2). The number of exons in MKKs ranged from one (HaMKK9, HaMKK4, and HaMKK5) to 12 (HaMKK3), with an average of 6.25 exons per gene (Table 2, Figure S3).

### 3.3. Phylogenetic Analyses

Full-length amino acid sequences of MPKs and MKKs of sunflower, *Arabidopsis* and soybean were employed for evaluating evolutionary relationships, as well as for nomenclature of the MPKs and MKKs of species under study. These sequences were subjected to multiple sequence alignment and subsequent phylogenetic analyses. Phylogenetic analyses included MPK and MKK gene sequences from, *A. coerulea*, *A. thaliana*, *A. trichopoda*, *C. reinhardtii*, *D. carota*, *G. max*, *H. annuus*, *O. sativa*, *S. lycopersicum*, and *V. vinifera.*

#### 3.3.1. MPKs

Sunflower MPK (HaMPK) protein sequence length ranged from 349 to 588 amino acid (aa), except for HaMPK4, which was only 157 aa. The average length of MPKs was 425 aa, with isoelectric points ranging from 5.22 (HaMPK13-1) to 9.65 (HaMPK23-1) and a predicted average molecular mass of 48523.772 Da (Table 1). Twenty-eight HaMPKs identified in this study were nested into four clades (A–D; each with bootstrap support > 70%) (Figure S4), which corresponded to their homologs in *A. thaliana* and *G. max*, except for the Clade C MPK members (Table S3). The Clade A members in this study include the previously identified group A and B members of *A. thaliana* MPKs [3,4]. Likewise, Clade B consists of previously identified group C members of *A. thaliana* MPKs. In addition, Clade C includes the members identified in group E of soybean MPKs [26]. The number of HaMPKs in Clades A, B, C, and D were nine, four, five, and ten, respectively. Sunflower MPK Clade C included five members with HaMPK22 (a homolog to GmMPK22-1 and GmMPK22-2) and HaMPK23-1/23-2/23-3/23-4 (homologs to the corresponding GmMPK23-1/23-2/23-4/23-4). Clade A and B consisted of members with phosphorylation motif TEY (except for HaMPK23-1 and HaMPK23-2 that are nested within Clade C), while those with the TDY motif were found in Clade C and D. The sunflower MPK orthologs are shown in Table S4. The phosphate-binding P-loop, the catalytic C-loop, D(L/I/V)K, and the activation- or T-loop, TxY, in MPKs were defined as (I/V/L)GxGx(S/F/G)GxV, HRD(L/I)KPxN and T(D/E)Y in sunflower, respectively. The protein sequence of HaMAPK23-3 had a variation in catalytic C-loop, D(L/I/V)K motif, as it possessed ‘Phenylalanine (F)’ instead of ‘Leucine/ Isoleucine/Valine (L/I/V)’. Other additional motifs, such as VAIKKIxxxF, were defined as VA(I/V/M)KK(I/M)xxx(F/Y) in the protein sequences of MPKs. The MPKs that belonged to Clade C possessed VA(I/V/M)KKMxxxY. The motifs ‘DFGLAR’ and ‘TRWYRAPE’ were found conserved in all of the MPKs of sunflower. HaMPK4 was the only member that lacked phosphate binding P-loop and VAIKKIxxxF motif. The structural analyses mapped onto phylogeny provided important insights into the duplication events. In the HaMPK gene family, the number of introns ranged from one (HaMPK4) to 17 [three members from Clade C (HaMPK22, HaMPK23-4/23-2)]. The gene members showed a similar pattern of exon/intron structure within the clades. The majority of the HaMPKs (seven) in Clade A consist of six exons, and members, HaMPK13-1 and HaMPK4 had seven and two exons, respectively. In Clade B, all three members consisted of three exons. Three of the five members in Clade C possessed 18 exons, and HaMPK23-1 and HaMPK23-3 possessed 15 and 16 exons, respectively. Likewise, half of the gene members in Clade D (five) possessed ten exons, two (HaMPK19-2 and HaMPK18) possessed nine exons, and three genes (HaMPK8, HaMPK15, and HaMPK9-1) possessed 11 exons (Figure S1).

**Figure 3:**
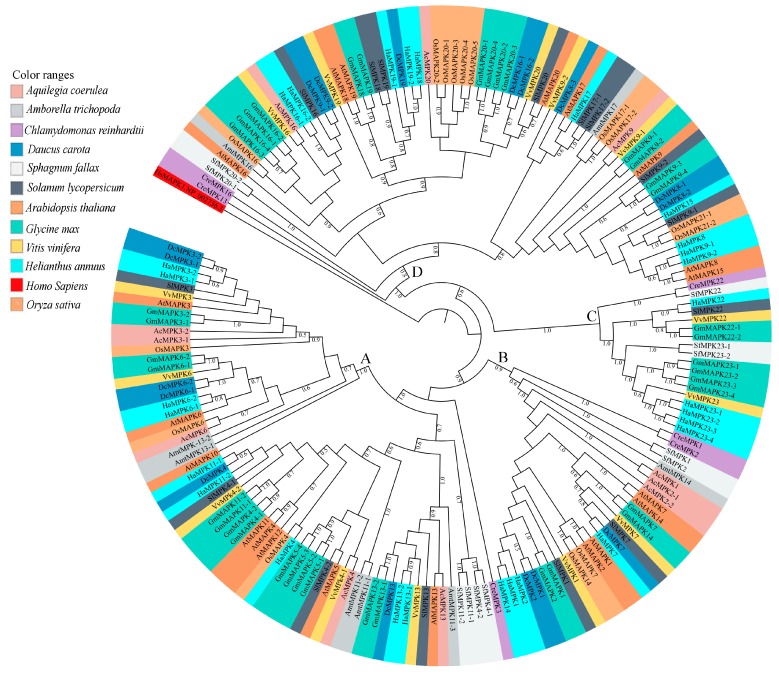
Maximum Likelihood (ML) tree constructed using full length amino acid sequences from *Amborella trichopoda* (Amt), *Arabidopsis thaliana* (At), *Aquilegia coerulea* (Ac), *Chlamydomonas reinhardtii* (Cre), *Daucus carota* (Dc), *Glycine max* (Gm), *Helianthus annuus* (Ha), *Oryza sativa* (Os), *Solanum lycopersicum* (Sl), and *Sphagnum fallax* (Sf), and *Vitis vinifera* (Vv) MPK proteins. Phylogenetic analysis with 100 bootstrap replicates was performed in the program MEGA7. *Homo sapiens*, HsMAPK1 (GenBank: NP_002736.3) was used as an outgroup. Different species are color-coded, and the MPK clades are labeled A–D. The Clade A members include the previously identified group A (MPK3, MPK6, MPK10) and B (MPK4, MPK5, MPK11, MPK12, MPK13) members of *A. thaliana* MPKs [3,4]. The Clade B members include the previously identified group C (MPK1, MPK2, MPK7, and MPK14) members of *A. thaliana* MPKs [3,4]. The Clade C members include the previously identified group E (MPK22 and MPK23) members of soybean MPKs [26]. The Clade D members include the previously identified group D (MPK8, MPK9, MPK16, MPK17, MPK18, MPK19, MPK20, and MPK21) members of *A. thaliana* MPKs [3,4].

**Figure 4:**
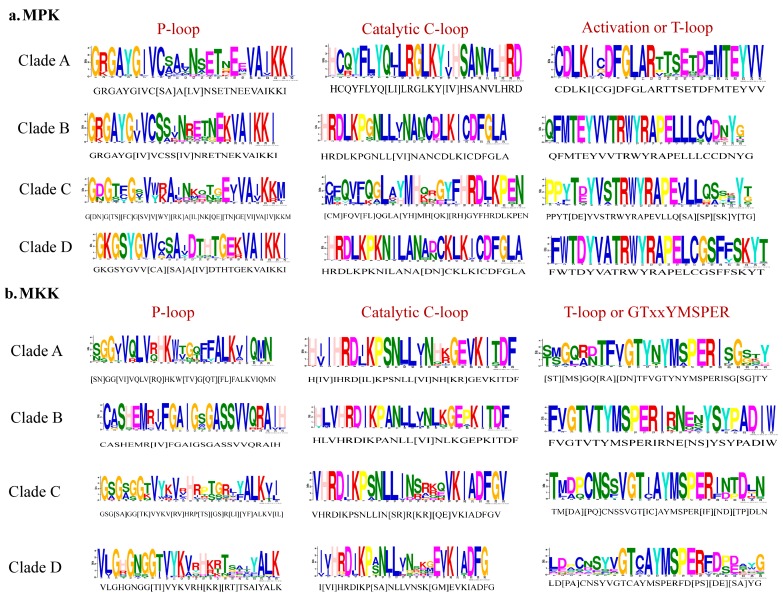
P-loop, catalytic C-loop, and activation or T-loop motifs representing variations in Clades A–D. (**a**) MPK; (**b**) MKK.

Phylogenetic analysis of full-length protein sequences was conducted to study evolutionary patterns of the MPKs in ten plant species with sequences of *C. reinhardtii* (Figure 3). The MPKs were nested in four clades (Clade A–D; Table S3). Clade A is the second largest clade, consisting of 64 MPKs of MPK3/6/4/11/5/13/10 of all species under the study. Clade B consists of 29 MPKs of MPK1/2/7 and 14. In the cases of *S. lycopersicum* and *V. vinifera*, these two species contain MPK1 and MPK7 in Clade B. Thus, MPK2 and MPK14 are absent in two species, but not only MPK2. In addition, *A. trichopoda* only has AmtMPK14 in Figure 3. Therefore, MPK1/2/7 of *A. trichopoda* is absent. The MPK14 of *V. vinifera* and *D. carota*, MPK2 of *S. lycopersicum* and *V. vinifera*, and MPK7 of *A. trichopoda* are absent. The smallest clade, Clade C, consists of 18 members of MPK22 and MPK23 from *H. annuus*, *G. max*, *S. lycopersicum*, *V. vinifera*, *S. fallax*, and *C. reinhardtii*. All the members of Clade A and B consist of the TEY motif, whereas some members of Clade C (HaMPK23-1/23-4, GmMAPK23-1/23-2/23-3/23-4, and VvMPK22) consist of the TEY motif. The largest clade, Clade D, consists of 70 MPKs of MPK16/18/19/20/21/17/9/8/15, and MPK13 of *C. reinhardtii*. All clades had moderate to strong support (bootstrap values ranging from 80 to 100%). Figure 4a and Supplementary File S3 show the motifs related to the P-loop, catalytic C-loop, and activation or T-loop, representing variations in clades A–D, including other predicted conserved domains of MPK group proteins. In addition, the clade divergence was also based on the common docking site, which is important for downstream target proteins. Clade A consisted of K-M-L-V-F-D-P-N-K-R-I-V-E-E-A-L, Clade B consisted of K-M-L-V-F-D-P-S-K-R-I-S-V-T-E-A-L, Clade C consisted of S-L-C-S-W-D-P-C-K-R-P-T-A-E-E-A-L, and Clade D consisted of R-L-L-A-F-D-P-K-D-R-P-T-A-E-E-A-L consensus common docking sites (Table 3).

#### 3.3.2. MKKs

Sunflower HaMKK protein sequence length ranged from 308 to 520 aa. The average length of proteins for MKKs was 372 aa, with isoelectric points ranging from 5.43 (HaMKK2) to 9.25 (HaMKK5) and a predicted average molecular mass of 42688.86 (Table 1). Corresponding with their homologs in *Arabidopsis* and *G. max*, the eight identified HaMKKs are divided into four distinct clades (Figure S5). The MKK homologs of MKK1/2/6-1/6-2/3/4/5/9 were only found in sunflower. The clades’ divergence followed serine/threonine amino acid motif patterns in sunflower. For example, Clade A contained SxxxxxS/TxxxxxT, Clade B with SxxxxxTxxxxxT, Clade C with SxxxxxTxxxxxS, and D with SxxxxxSxxxxxT. The HaMKKs in Clades A, B, C, and D were four, one, two, and one, respectively (Table S5). The orthologs of identified MKKs of sunflower in different plant species are represented in Table S6. In the HaMKK gene family, the number of introns ranged from zero (HaMKK9, HaMKK4, and HaMKK5) to 11 (HaMKK3) (Table 2, Figure S3). Clade A members HaMKK6-1 and HaMKK6-2 consisted of eight exons and are paralogs to each other. The remaining Clade A members, HaMKK2 and HaMKK1, consisted of nine and ten exons, respectively. The only member of Clade B, HaMKK3, consisted of twelve exons. Interestingly, the members of Clade C and D (HaMKK9, HaMKK4, and HaMKK5) had no introns.

**Figure 5:**
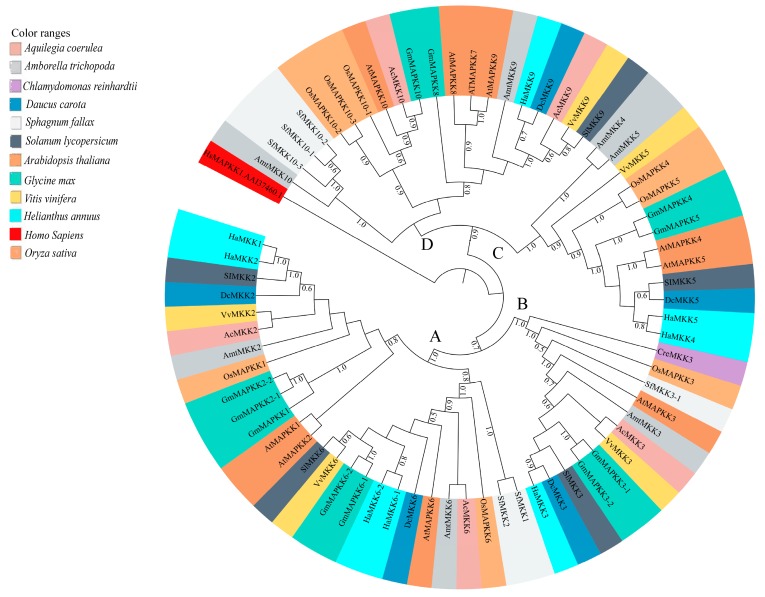
Maximum Likelihood (ML) tree constructed using full-length MKK amino acid sequences from *Amborella trichopoda* (Amt), *Arabidopsis thaliana* (At), *Aquilegia coerulea* (Ac), *Chlamydomonas reinhardtii* (Cre), *Daucus carota* (Dc), *Glycine max* (Gm), *Helianthus annuus* (Ha), *Oryza sativa* (Os), *Solanum lycopersicum* (Sl), and *Sphagnum fallax* (Sf), and *Vitis vinifera* (Vv). Phylogenetic analysis with 100 bootstrap replicates was performed in the program MEGA7. *Homo sapiens*, HsMAPKK1 (GenBank: AAI37460.1) was used as an outgroup. Different species are color-coded, and the MKK clades are labeled A–D. Clade A, B, C, and D members include the previously identified group A (MKK1, MKK2, and MKK6), group B (MKK3), group C (MKK4, MKK5), and group D (MKK7, MKK8, MKK9, and MKK10) members of *A. thaliana* MKKs, respectively [3,4].

Phylogenetic analysis of full-length MKK amino acid sequences from the plant species with sequences of *C. reinhardtii* under this study revealed four distinct clades (Clades A–D, Figure 5). Figure 4b and Supplementary File S4 show the motifs related to P-loop, catalytic C-loop, and activation or GTxxYMSPER, representing variations in Clades A–D, including other predicted conserved domains of MKK group proteins. The largest clade, Clade A, consisted of 26 MKKs, belonging to MKK1, MKK2, and MKK6. While MKK3 orthologs formed Clade B, consisting 12 MKKs, MKK4 and MKK5, with 16 members, formed Clade C. Gene MKK4 is absent in *S. lycopersicum*, *V. vinifera*, *D. carota*, and *C. reinhardtii* species. MKK7, MKK8, MKK9, and MKK10 formed Clade D, which consisted of 16 of the total MKKs under study. With respect to all MKKs belonging to ten species, the phosphate-binding P-loop, the catalytic C-loop, D(L/I/V)K, and activation- or T-loop, (S/T)xxxxx(S/T) varied across the clades. The GTxxYMSPER motif was well conserved in all species except for the OsMAPKK6 and AmtMKK6 with GTxxYMAPER in Clade A and OsMAPKK10-1 in Clade D with GTxxYMSPEK. The ATP binding signature in MKK of sunflower terminates with ALK except for GmMAPKK6-1 (completely absent), CrMKK3 with AVK, VvMKK4 with ANT, OsMAPKK10-1 (completely absent), and OsMAPKK10-1 with AVK. The Timetree based on the 11 MKK3 (each MKK3 protein from all species belonging to Clade B) sequences shows the evolutionary divergence across all species under study. Upon use of three constraints of divergence between *O. sativa* and *A. trichopoda* (168–194 MYA), *G. max* and *H. annuus* (110–124 MYA), and *V. vinifera* and *A. thaliana* (105–115 MYA), the approximate divergence of these MKK3 proteins across species has been found. For instance, DcMKK3 and SlMKK3 diverged 90.70 MYA from HaMKK3 (Figure S6).

### 3.4. Expression Analysis and miRNA Prediction of Sunflower MPKs and MKKs

**Figure 6:**
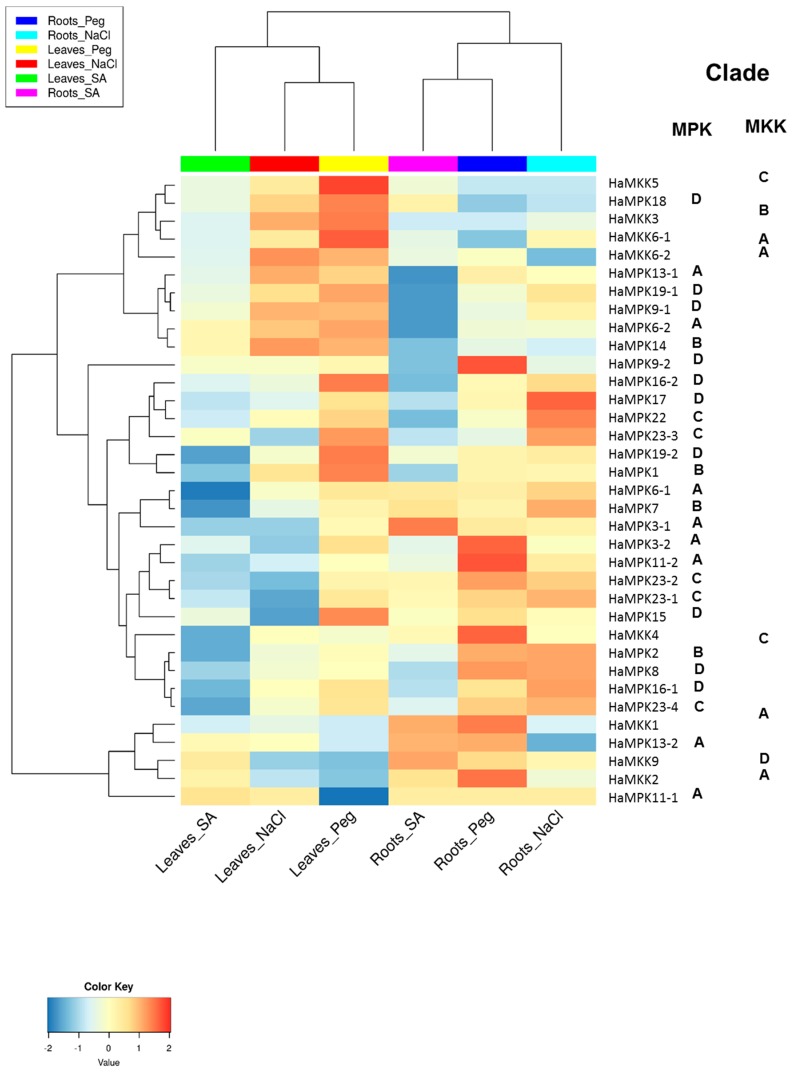
Expression profile of sunflower MPK and MKK genes visualized as a heatmap, with clade information. The heatmap was generated using log_2_FC values. The expression pattern is in response to salicylic acid (SA), salt (NaCl) and polyethylene glycol (Peg) in leaves and roots. The RNA-seq data was accessed from NCBI SRA SRP092742 [SRR4996815 (Roots Peg), SRR4996819 (Roots_NaCl), SRR4996823 (Leaves_Peg), SRR4996828 (Roots_Control), SRR4996834 (Leaves_NaCl), SRR4996836 (Leaves_Contol), SRR4996839 (Leaves_SA), and SRR4996847 (Roots_SA)].

The functional analysis of both HaMPKs and HaMKKs was studied using RNA-seq data available in NCBI. Since the sunflower genome was recently available, the expression data for pathogen stress were not available in the public databases. We investigated the expression pattern of MPKs and MKKs in leaves and roots treated with one hormone treatment (SA) and two abiotic stresses (NaCl and Peg). We did observe expression patterns for all HaMPKs and HaMKKs except for HaMPK4 (Supplementary File S5). The *k*-means clustering result showed that the HaMPKs and HaMKKs were clustered into four groups (Figure S7 and Table S7). Cluster A consisted of seven HaMPKs (from Clades A, B, and D) and four HaMKKs (from Clades A, B, and C). Cluster B consisted of three HaMKK genes (from Clades A and D) and two HaMPK genes (from Clade A). Cluster C consisted of three genes belonging to both HaMPKs (from Clades A and D) and one HaMKK (from Clade C). Cluster D consisted of 15 genes belonging to HaMPKs (belonging to clades A–D). The log_2_FC for each gene and hierarchical clustering of HaMPKs and HaMKKs representing the functional divergence of these genes are represented in Figure S8 and Figure 6, respectively. Some genes were upregulated in response to the treatments compared to the control of their respective tissues. For instance, in leaves, HaMKK5, HaMKK6-2, HaMPK3-2, HaMPK11-1, HaMPK14, HaMPK1, HaMPK6-2, HaMPK19-1, and HaMPK18 showed log_2_FC > 1 in response to Peg; HaMKK5, HaMKK6-2, HaMPK11-1, HaMPK14 showed log_2_FC > 1 in response to NaCl; HaMPK11-1 showed log_2_FC > 1 in response to SA. In roots, HaMKK4, HaMKK1, HaMKK2, HaMPK3-2, HaMPK13-2, HaMPK23-2, HaMPK9-2 and HaMPK11-2 showed log_2_FC > 1 in response to Peg; HaMKK9, HaMPK13-2, HaMPK6-1, and HaMPK3-1 showed log_2_FC in range of 0.7 to 1.45 in response to SA; HaMPK6-1, HaMPK2, HaMPK23-2, and HaMPK17 showed log_2_FC > 0.9 in response to NaCl. In contrast, some genes were downregulated in response to the treatments compared to the control of their respective tissues. For example, in leaves, HaMKK9, HaMKK2, and HaMPK13-2 showed log_2_FC in a range of −0.6 to −0.8 in response to Peg; HaMKK9, HaMPK7, HaMPK23-1 showed log_2_FC in a range of −0.6 to −0.8 in response to NaCl; HaMKK4, HaMPK7, and HaMPK11-2 showed log2fold change in a range of −0.58 to −2.11 in response to SA. Likewise, in roots, HaMPK14 showed log_2_FC of −0.53 in response to Peg; HaMKK6-2, HaMPK13-2, HaMPK14, and HaMPK9-2 showed log2fold change in a range of −0.62 to −1.50 in response to NaCl; HaMPK14, HaMPK19-1, and HaMPK9-2 showed log_2_FC in a range of −0.68 to −1.6 in response to SA. In addition, the expression of HaMPKs, HaMKKs showed functional divergence in response to stresses as the clustering of these genes in a heatmap was not in accordance with the nesting pattern within clades in phylogenetic trees. The potential miRNA target sites in MPKs and MKKs identified using psRNATarget server revealed five (han-miR156a/b/c, han-miR160a, han-miR3630-5p) of seven miRNA families that may be involved in targeting sunflower MPKs only (Table S8). HaMPK16-2, HaMPK11-1, and HaMPK23-3 were found to be targeted by both miRNAs (han-miR156a/b).

### 3.5. Tajima’s Relative Rate and Neutrality Tests on MPKs and MKKs

Separate statistical analyses were performed selecting three random sequences from MPKs and MKKs group. For Tajima’s relative rate test for MPKs and MKKs, the sequences were selected from the species representing a diverse taxonomic group: monocot, a dicot, basal angiosperm, bryophytes, and algae. For the analysis of MPK genes following a group of sequences were selected: (a) OsMAPK4 (monocot) and HaMPK6 (dicot) with AmtMPK13-1 (basal angiosperm); (b) OsMAPK4 (monocot) and HaMPK6 (dicot) with sequence SfMPK4-1 (bryophyte); and (c) OsMAPK4 (monocot) and HaMPK16-1 (dicot), with sequence CreMPK2 (algae) (Table S9). The plant group combination in column 1, 2 and 3 of MPKs resulted in *p*-values of 0.01, 0.0053, and 0.0007, with χ^2^ values of 6.54, 7.78 and 11.46, respectively. For MKKs, the following group of sequences were selected: (a) OsMAPKK5 (monocot) and HaMKK6-1 (dicot) with AmtMKK6 (basal angiosperm); (b) OsMAPKK5 (monocot) and HaMKK6-1 (dicot) with sequence SfMKK3 (bryophyte); and (c) OsMAPKK5 (monocot) and HaMKK6-1 (dicot) with CreMKK3 (algae) (Table S10). The plant group combination in columns 1, 2 and 3 of MKKs resulted in *p*-values of 0, 0.04965 and 0.05687 with χ^2^ values of 100.55, 3.85 and 3.36, respectively. Tajima’s Relative Rate test is commonly used to analyze variation in both DNA and amino acid sequences [78]. This test has been applied to various genes belonging to different gene families, such as MAPKs and WRKY transcription factors [1,78]. In this study, the *p*-value (less than 0.05) and χ^2^ statistic showed randomly selected sequences of MPKs and MKKs of different plant groups to be statistically significant, rejecting the null hypothesis of equal rates between selected sequences of different plant groups. The interpretation of Tajima’s D is as follows: D = 0 (observed variation is similar to the expected variation, which shows evidence of no selection), D < 0 (presence of excessive rare alleles, suggesting recent selection sweep and recent population expansion), and D > 0 (lack of rare alleles, suggesting balanced selection and sudden population contraction) [72,73]. The values in the ranges greater than 2 or less than −2 are considered to be statistically significant [72,73]. In our study, Tajima’s neutrality test statistics (D) were found to be 5.391062 for MPKs and 5.928839 for MKKs (Table S11). This suggests that both MPKs and MKKs have undergone a balanced selection with contraction in gene family size. Also, the average heterozygosity of both MAPKs and MKKs is more than those of the segregating sites, suggesting a high frequency of polymorphism.

## 4. Discussion

MAPK signaling in plants plays important roles in multifaceted biological processes such as growth, development, and regulation of various environmental stresses [4,34,36,79,80,81,82,83,84,85,86,87,88,89,90]. The MPK and MKK genes have been strong candidates for studying the evolution of gene families in plant species as well [27,28,39,91]. In this study, the HMM analysis of protein sequences and examination of the signature motifs resulted in the identification of 96 MPK and 37 MKK genes in *A. coerulea*, *A. trichopoda*, *C. reinhardtii*, *D. carota*, *H. annuus*, *S. fallax*, and *S. lycopersicum*.

### 4.1. Nomenclature of MPKs and MKKs

A recent study on various Triticeae species (wheat, barley, rye, and triticale) by Goyal et al. 2018 [35] reported numerous discrepancies in MAPK nomenclature of wheat and barley and suggested a new name based on sequence homology. A consistent nomenclature of proteins belonging to the same gene family across species based on orthology facilitates easy prediction and understanding of the function of a particular protein [92]. Cakir and Kılıçkaya 2015 [37] reported MAP kinase cascade genes in *V. vinifera* and confirmed the orthology of VvMPK14, VvMPK12, VvMPK11, VvMPK13, VvMPK7, VvMPK3, VvMKK5, VvMKK3, and VvMKK2 to *Arabidopsis* AtMPK6, AtMPK3, AtMPK13, AtMPK12, AtMPK16, AtMPK9, AtMKK3, AtMKK6, and AtMKK2, respectively. Likewise, MAP Kinase cascade genes analyses in *Ziziphus jujuba* [30] provided nomenclature of MAP kinase cascade genes based on the order of appearance in different groups in the phylogenetic tree, and not based on orthology (or sequence homology) to *Arabidopsis* MAP Kinase cascade genes. The proper nomenclature of these MAP Kinase cascade genes should be used following an orthology or sequence homology-based MAPK gene nomenclature guidelines to maintain consistency across the plant kingdom.

### 4.2. Diversity and the Phylogenetic Relationship of MPKs

Our identification of MPKs yielded a slight variation in the number of genes from the previous studies; for example, we identified 15 MPKs in *S. lycopersicum*, which is different from Kong et al. 2012 [93], who reported 16 MPKs, and Mohanta et al. 2015 [1], who found 17 MPKs in the tomato genome. The number of AcMPKs in this study was 11, whereas Mohanta et al. 2015 [1] reported only 10 AcMPKs. In *C. reinhardtii*, six CreMPKs identified in this study were consistent with Mohanta et al. 2015 [1], whereas Dóczi et al. 2012 [39] reported only five CreMPKs. The variation in several genes within the same species in different studies might come as a result of different statistical and stringency parameters employed during HMM profiling and further downstream motif analysis. The detailed study of MPKs of *D. carota*, *A. trichopoda*, *S. fallax*, and *H. annuus* has never been reported in previous studies. The number of MPK genes in sunflower is higher than that previously identified in numerous other plant species, such as *Arabidopsis* (119Mb) [3] and rice (420Mb) [94], and lower than in soybean (1100Mb) [26]. Even the size of the sunflower genome, which is believed to have undergone the first whole genome triplication approximately 38–50 MYA, and whole genome duplication approximately 29 MYA, is about 3.5 times larger [95] than that of the soybean genome: the number of MPKs is lower in sunflower than soybean. Soybean has undergone two polyploidization events, approximately 59 and 13 MYA [75,96]. Thus, recent polyploidy in plants has resulted in extra copies of genes to their genome [97,98]. The slightly lower number of MPKs in sunflower might be due to past polyploidization events and the recent amplification of repetitive elements causing highly similar and related sequences [99]; the sunflower genome also encodes 52,243 proteins [42], which is slightly fewer than the soybean genome (56,044 proteins) [75].

Phylogenetic analysis of HaMPKs revealed four distinct clades, which were consistent to the MPKs previously identified in *Arabidopsis* [100], poplar [101], rice [102], *Brachypodium distachyon* [33], *Malus domestica* [32], *Ziziphus jujuba* [30], Triticeae species [35], *Brassica rapa* [28], and *Fragaria vesca* [103]. In Clade A, sunflower has one extra copy of MPK3, MPK6, MPK11, and MPK13 genes that might be because of duplications after the divergence from *Arabidopsis*. Such extra copies of these genes have also been observed in soybean [26]. The two copies of MPK3 and MPK6 were also found in *D. carota*. The nesting pattern of sunflower and other species’ MPK genes with the characterized *Arabidopsis* MPKs suggest their potential role in respective functions. AtMPK3 is involved in various signaling pathways related to various stresses, such as wounding and hypersensitive responses elicited by Avr-R gene interaction [8,104]. The MAP kinase genes, IbMPK3 and IbMPK6, in sweet potato (*Ipomoea batatas*), and homologs of AtMPK3 and AtMPK6, provide resistance to *Pseudomonas syringae* pv. *tabaci* (*Pta*) bacteria in tobacco leaves, and were induced in various abiotic stresses, as well [84]. In maize, ZmMPK3, a homolog of AtMPK3 is induced in response to various environmental stresses [105]. Similarly, AtMPK4 and AtMPK6 are involved in response to abiotic and biotic stress such as cold, drought, touch and wounding, resulting in the production of reactive oxygen species in *Arabidopsis* [106,107]. AtMPK4 is phosphorylated and activated by the upstream components AtMEKK1 and AtMKK2 upon cold and salt stress signaling in *Arabidopsis* [107,108]. Clade A also consists of AtMPK5, the homolog of which in rice, OsMPK5, is well characterized to regulate stress responses [109]. All copies of MPK1/2, MPK7/14 are retained in soybean in sunflower, soybean, and *Arabidopsis*. Among them, AtMPK1, AtMPK2, AtMPK7, AtMPK14 are phosphorylated by AtMKK3 upon abscisic acid application in *A. thaliana* plantlets [110]. AtMPK1 is induced upon salt stress, whereas some MPKs in rice and alfalfa such as BWMK1 and TDY1, respectively, are activated upon wounding by pathogens [111,112,113]. *G. max* MAP kinase 1 (GMK1), a homolog of AtMAPK1, is activated in response to salt stress in soybean [114]. Likewise, a homolog of AtMPK7 in maize, ZmMPK7 is involved in the removal of reactive oxygen species upon induction by abscisic acid and hydrogen peroxide in maize [115]. Another homolog of AtMPK1 in *Hordeum vulgare* (HvMPK4) showed enhanced resistance to *Magnaporthe grisea* and enhanced tolerance to salt stress [85]. Clade C members include the homologs of *G. max* GmMAPK22-1/22-2 and GmMAPK23-1/23-2/23-3/23-4 [26] with no MPKs in *Arabidopsis*. A single copy of GmMAPK22-1/GmMAPK22-2 ortholog is retained in sunflower, and hence it is named HaMPK22. Meanwhile, all copies of GmMAPK23-1/23-2/23-3/23-4 are retained in sunflower and are hence named HaMPK23-1/23-2/23-2/23-3/23-4. All the members of Clade D consist of the TDY motif in the T-loop and are homologs to various *Arabidopsis* and soybean MPKs belonging to MPK16/19/18/8/15/17/9.

Gene members HaMPK3-1/3-2, HaMPK6-1/6-2, HaMPK9-1/9-2, HaMPK11-1/11-2, HaMPK13-1/13-2, HaMPK16-1/16-2, HaMPK19-1/19-2, and HaMPK23-2/23-4 are present on different chromosomes, while only paralogs HaMPK23-1/23-3 are present on the same chromosome 3. Other MPKs, such as AcMPK3-1/3-2, AcMPK2-1/2-2, DcMPK3-1/3-2, DcMPK6-1/6-2, DcMPK8-1/8-2/8-3, DcMPK9-1/9-2, SfMPK4-1/4-2, SfMPK20-1/20-2, SfMPK23-1/23-2, SlMPK4-1/4-2, SlMPK17-1/17-2, SlMPK9-1/9-2, AmtMPK13-1/13-2, are present on different chromosomes. The only AmtMPK11-1/11-2 pair is present in the same scaffold (AmTr_v1.0_scaffold00001) (Table S1). This suggests a potentially crucial role of segmental duplications and transposition events in the evolution of MAPKs in sunflower and other plant species, except for the HaMPK23-1/23-3 and AmtMPK11-1/11-2 pairs, in which tandem duplication might have been involved. Such features of segmental and tandem duplications in MPKs are also evidently seen in many plant species such as soybean [26], apple [32], cotton [116]. Such duplications are the major reason for the expansion of the many gene families, such as nucleotide-binding site-leucine-rich repeat (NBS-LRR), cytochrome P450 family, transcription factors and many more [117].

### 4.3. Diversity and Phylogenetic Relationship of MKKs

Sunflower MKKs also formed four distinct clades (A–D) with previously identified MKKs of *Arabidopsis* and soybean. These four clades (A–D) are consistent with the MKKs of various plant species such as *Arabidopsis* [100], rice [102], poplar [101], *B. distachyon* [33] and apple [32]. MKK clades consist of well-characterized MKK proteins such as AtMKK1/2/3/4/5 [118,119,120,121]. Clade A consists of HaMKKs grouped with AtMKK1/6/2, GmMAPKK6-1/6-2, GmMAPKK1, GmMAPK2-1/2-2. Sunflower and soybean have extra one copy of MKK6 compared to *Arabidopsis* and other plant species under study, including *S. fallax*. This suggests that the extra one copy of MKK6 was not seen until soybean diverged from *Arabidopsis*. Also, the retention of at least one copy of MKK6 in all species suggests its important role in signaling mechanisms during various stresses. We did not find a copy of MKK2-2 in sunflower, as is found in soybean (GmMAPKK2-2). The characterized AtMKK1 protein (orthologue of HaMPKK1) is induced upon the application of various stresses such as wounding, drought, cold, and high salinity in *Arabidopsis* seedlings [118]. AtMKK2 (ortholog of HaMKK2) is activated upon cold and salt stress signaling in *Arabidopsis* and mediates the phosphorylation of downstream MPKs [107]. Clade B consists of MKKs from the MKK3 proteins across all species under study, including *C. reinhardtii*. All species have a single copy of the MKK3 proteins except *G. max*, with two copies (GmMAPKK3-1/3-2). Two copies of MKK3 proteins in soybean is expected, as they underwent two duplication events to become a tetraploid. A divergence-time estimation based on Clade B sequences (each from all species) revealed how MKK3 proteins are conserved and retained in Algae, Bryophyte, Amborellales, Monocots, Ranunculales, Rosids, and Asterids. The divergence time analysis of MKK3 with CreMKK3 as the outgroup showed bryophyte and Amborellales being sister to the land plants and other extant species, which is consistent to previous studies [122,123] and follows the evolutionary history inferred on the Angiosperm Phylogeny Website [124]. One of these MKK3s, AtMKK3 is activated upon exposure to various stresses, such as cold, salt, hyperosmotic and ABA treatments [120]. This suggests the potential role of HaMKK3 in such stresses. Clade C consists of both copies of AtMKK4 and AtMKK5 only in *A. trichopoda*, *O. sativa*, and sunflower. However, *V. vinifera*, *S. lycopersicum*, and *D. carota* consist copies of MKK5 (MKK4 group absent). AtMKK4 and AtMKK5 are activated in *Arabidopsis*, mediating cell death and production of hydrogen peroxide [119]. In Clade D, the orthologs for MKK9 were found in all angiosperms except in soybean and *O. sativa*. Interestingly, we found three copies of MKK10 in *S. fallax*, as in *O. sativa*, and one copy of MKK10 in basal angiosperm, *A. trichopoda,* and Ranunculales, *A. coerulea.* We did not find any copy of MKK10 in sunflower, *S. lycopersicum*, *D. carota*, or *V. vinifera*. We observed HaMKK4/5/9 with one exon each that correlates to the At1g51660 (AtMKK4), At3g21220 (AtMKK5), and At1g73500 (AtMKK9), consisting of one exon per gene (https://www.Arabidopsis.org/index.jsp). Also, members belonging to Clade C and D in *Gossypium raimondii* had one exon in each [116]. This suggests that gene members belonging to Clade C and D encode proteins that are well conserved across plant species. Altogether, the diversity in the exon-intron structures might imply that duplication events caused the evolution of these genes under different environmental conditions. Also, AtMKK1 and AtMKK2 are involved in maintaining ROS homeostasis in *Arabidopsis* [121]. Since the paralog pairs, HaMKK6-1/6-2 and SfMKK10-1/10-2, are present on their different respective chromosomes, we infer a possible role of segmental duplications.

### 4.4. Expression Analysis and miRNA Prediction

In this study, we explored the expression pattern of MPKs and MKKs of sunflower under one hormone treatment, SA and two simulated abiotic stresses: NaCl for salinity, and Peg for osmotic stress in leaves and roots from the publicly available RNA-seq data. The expression of all sunflower MPKs and MKKs was detected in both leaves and roots, except for HaMPK4. In response to hormone SA, HaMPK11-1 was upregulated in leaves; HaMKK9, HaMPK13-2, HaMPK6-1, and HaMPK3-1 were upregulated in roots; HaMKK4, HaMPK7, and HaMPK11-2 were downregulated in leaves; HaMPK19-1, HaMPK14, and HaMPK9-2 were downregulated in roots. It has been established that SA is directly involved in MAPK phosphorylation [125]. SA-induced protein kinase (SIPK; AtMPK6) and wound-induced protein kinase (WIPK; AtMPK3) are important in balancing salicylic acid or jasmonic acid during herbivore wounding [126]. In *Arabidopsis*, AtMKK9 and AtMPK6 play important role in leaf senescence, which is a complex process caused by various factors including salicylic acid [127]. Also, ZmMPK3 in *Zea mays* is activated upon the application of SA hormone [128]. Thus, HaMPK3-1, HaMKK9, and HaMPK6-1 might play an important role in leaf senescence and salicylic acid pathways in sunflower. In response to NaCl, HaMKK5, HaMKK6-2, HaMPK11-1 were upregulated in leaves; HaMPK14, HaMPK6-1, HaMPK2, HaMPK23-2, and HaMPK17 were upregulated in roots; HaMKK9, HaMPK7, HaMPK23-1 were downregulated in leaves; HaMKK6-2, HaMPK13-2, HaMPK14, and HaMPK9-2 were downregulated in roots. Among them, HaMPK17 play an important role under salinity stress, as its ortholog in *Gossypium hirsutum*, GhMPK17, was induced by salt, osmosis and abscisic acid [129]. The expression pattern of some genes depended on different parts of the plant, for example, HaMKK6-2 was upregulated in leaves and downregulated in roots in response to NaCl. In response to Peg, HaMKK5, HaMKK6-2, HaMPK3-2, HaMPK11-1, HaMPK14, HaMPK1, HaMPK6-2, HaMPK19-1, and HaMPK18 were upregulated in leaves; HaMKK4, HaMKK1, HaMKK2, HaMPK3-2, HaMPK13-2, HaMPK23-2, HaMPK9-2, and HaMPK11-2 were upregulated in roots; HaMKK9, HaMKK2, and HaMPK13-2 were downregulated in leaves; HaMPK14 was downregulated in roots. This reveals that at least 19 HaMPK and seven MKK genes were induced upon these treatments, as compared to the control. Among them, some genes are induced upon multiple treatments. For example, HaMKK4 and HaMKK6-2 were induced upon both NaCl and Peg; HaMPK6-1 was induced upon NaCl and Peg; HaMPK16-2 was induced upon both SA and NaCl. The functional divergence can be observed on both HaMPKs and HaMKKs, as the hierarchical clustering patterns of expression of these genes do not follow the nesting pattern within clades in the phylogenetic trees, except for a few genes. For example, in MPKs, HaMPK22/23-3 that belonged to Clade C, HaMPK3-1/3-2/11-2 that belonged to Clade A, and HaMPK9-2/16-2/17 that belonged to clade D showed hierarchical clustering for expression of these genes. However, only HaMKK6-1/6-2 that belonged to Clade A of the MKK subgroup showed hierarchical clustering for expression of these genes. This shows the functional divergence and convergence of the HaMPK and HaMKK genes within and among the clades under different stress responses. Among seven published *H. annuus* microRNAs, five families of miRNAs are involved in possibly targeting eight MPKs. We did not find any miRNAs targeting HaMKK genes. Previous studies have reported the role of miRNAs in MAPK signaling pathways of animal systems in chronic myeloid leukemia [130], papillary thyroid carcinoma [131], *Caenorhabditis elegans* [132]. Not only in animals, but studies also reported the prediction of miRNAs targeting MAPK genes of plants such as *Gossypium hirsutum* (ghr-miR5272a regulating MAPKK6) [133] and *Oryza sativa* (miR1429_5p targeting MPK17-1 and miR531 families targeting various MKKK transcripts) [134].

## 5. Conclusions

This study represents the first genome-wide identification, analysis and nomenclature of MPKs and MKKs in *H. annuus*, *D. carota* and, *S. fallax*, as well as reassessment of these genes in *A. coerulea*, *A. trichopoda, C. reinhardtii,* and *S. lycopersicum.* We identified 28 MPKs and eight MKKs in sunflower, and studied their genomic architecture, phylogenetic relationships, and functions in relation to nine other plant species (including *A. thaliana*, *G. max*, *O. sativa*, and *V. vinifera*). While the 3.6 gigabase sunflower genome is one of the largest among plants with available complete genome sequences, more MPKs and MKKs were found in soybean, which has a genome size of 975 Mbs. Analyses of P-loop, catalytic C-loop, and T-loop showed that HaMPKs and HaMKKs could be classified into four clades, which are comparable to those groups identified in *A. thaliana* and *G. max.* However, clades such as Clade A, B, and C of MPKs consisted of different group members of *A. thaliana* and *G. max.* Among the MPK and MKK genes studied, the MKK3 proteins were well- conserved and retained in all species included in this study, including the outgroup *C. reinhardtii*, which warrants further exploration of these proteins across a wide array of species. The transcriptome data generated under hormone and abiotic stress treatments revealed diverse expression patterns of sunflower MPKs and MKKs exhibiting a dynamic role in adaptation to changing environmental conditions. We observed functional divergence of the HaMPK and HaMKK genes within the gene members of the same clade. The results of this study are important for understanding diversity and evolution of the MAPK gene family in plants and enhancing our knowledge of MAPK signaling pathways in sunflower. These findings can inform cultivar improvement in sunflower through stress-tolerance breeding.

## Figures and Tables

**Table 1:** Abundance of MPK and MKK genes in the genomes of 11 species used in this study.

Plant Species	Ploidy	Size of Genome (Mbs) ^γ^	No. of loci ^γ^	MPK	MKK
*Amborella trichopoda* ^‡^	Diploid	706	26846	8	7
*Aquilegia coerulea* ^‡^	Diploid	302	24823	11	5
*Arabidopsis thaliana*	Diploid	135	27416	20 ^a^	10 ^a^
*Chlamydomonas reinhardtii* ^‡^	Haploid	111.1	17741	6	1
*Daucus carota* ^‡^	Diploid	421	32,113	17	5
*Glycine max*	Tetraploid	975	56044	38 ^b^	11 ^b^
*Helianthus annuus* ^‡^	Diploid	3600	52243	28	8
*Oryza sativa*	Diploid	372	39049	16 ^c^	8 ^c^
*Solanum lycopersicum* ^‡^	Diploid	900	34727	15	5
*Sphagnum fallax* ^‡^	Haploid/Diploid	395	26939	11	6
*Vitis vinifera*	Diploid	487	26346	14 ^d^	5 ^d^

^‡^ = Plant species with MPKs and MKKs identified or revisited in this study; ^γ^ = References on the size of genome and number of loci *Amborella trichopoda* [44], *Arabidopsis thaliana* [74], *Aquilegia coerulea* [45], *Chlamydomonas reinhardtii* [47], *Daucus carota* [41], *Glycine max* [75], *Helianthus annuus* [42], *Oryza sativa* [76], *Solanum lycopersicum* [43], and *Sphagnum fallax* [46], and *Vitis vinifera* [77]; a = [10], b = [26], c = [4], d = [37]

**Table 2:** Sunflower MPK and MKK genes with their proposed name, GeneID, chromosomal location (Chr), strand direction (Str), start and end position of the genes on chromosome, protein length (PL), number of exon (Exo) and intron (Int), subcellular localization [Sl; M = Mitochondria and C = Chloroplast, - = Subcellular locations other than mitochondria or the chloroplast), isoelectric points (pI) and molecular weight (Mw)].

Name	Gene ID	Chr	Str	Start	End	PL	Exo	Int	Sl	pI	Mw
**MPK**											
HaMPK6-1	HanXRQChr01g0023391	Ha1	-	130301686	130292965	359	6	5	-	5.85	41581.61
HaMPK16-1	HanXRQChr03g0071491	Ha3	-	77378137	77372246	564	10	9	-	9.17	64059.43
HaMPK7	HanXRQChr03g0074811	Ha3	+	102410161	102406169	353	3	2	-	7.62	40274.83
HaMPK23-1	HanXRQChr03g0081221	Ha3	+	129978443	129973452	453	15	14	-	9.65	50392.28
HaMPK23-3	HanXRQChr03g0081391	Ha3	+	130506162	130500013	423	16	15	-	8.91	47648.79
HaMPK22	HanXRQChr04g0108301	Ha4	-	77321727	77315970	432	18	17	-	5.46	49633.87
HaMPK11-1	HanXRQChr04g0121371	Ha4	+	158781451	158778221	358	6	5	M	6.42	41228.21
HaMPK3-1	HanXRQChr05g0133161	Ha5	+	21064225	21061089	358	6	5	-	5.68	41323.35
HaMPK8	HanXRQChr05g0143371	Ha5	-	116774638	116767923	505	11	10	-	6.8	57051.89
HaMPK2	HanXRQChr05g0151241	Ha5	-	169574750	169571609	349	3	2	-	6.54	40295.67
HaMPK11-2	HanXRQChr06g0167011	Ha6	-	7104659	7099870	359	6	5	M	6.25	41336.17
HaMPK4	HanXRQChr06g0170261	Ha6	+	16894292	16893100	157	2	1	M	8.36	17702.54
HaMPK13-1	HanXRQChr06g0175501	Ha6	-	34635251	34631528	363	7	6	-	5.22	41353.31
HaMPK9-1	HanXRQChr06g0183531	Ha6	+	90706107	90699312	478	11	10	-	6.53	54442.91
HaMPK23-4	HanXRQChr08g0226701	Ha8	+	84318787	84308381	442	18	17	-	9.52	49480.06
HaMPK15	HanXRQChr08g0227231	Ha8	+	87599490	87591577	501	11	10	-	8.53	57073.07
HaMPK3-2	HanXRQChr08g0229941	Ha8	-	101013127	101009864	358	6	5	-	5.58	41298.31
HaMPK13-2	HanXRQChr08g0230171	Ha8	-	102808229	102804252	362	6	5	-	5.85	41552.83
HaMPK14	HanXRQChr09g0243011	Ha9	+	34673154	34669292	362	3	2	-	5.57	41423.42
HaMPK16-2	HanXRQChr09g0248301	Ha9	+	76212398	76202758	559	10	9	-	9.07	63370.4
HaMPK1	HanXRQChr09g0269211	Ha9	-	185086347	185083825	361	3	2	-	6.64	41831.44
HaMPK19-2	HanXRQChr11g0330461	Ha11	+	43791321	43784989	574	9	8	-	9.33	65344.85
HaMPK6-2	HanXRQChr11g0343001	Ha11	-	125967866	125963374	359	6	5	-	5.8	41553.72
HaMPK19-1	HanXRQChr13g0389781	Ha13	-	19048315	19044532	588	10	9	-	9.06	66613.36
HaMPK23-2	HanXRQChr13g0411961	Ha13	-	142634442	142625511	459	18	17	-	9.63	50984.95
HaMPK9-2	HanXRQChr14g0432771	Ha14	-	49683290	49679650	484	10	9	-	6.57	55530.13
HaMPK17	HanXRQChr15g0484561	Ha15	-	84424855	84420653	429	11	10	-	6.24	49909.6
HaMPK18	HanXRQChr15g0495321	Ha15	-	160155012	160149273	563	9	8	-	9.47	64374.62
**MKK**											
HaMKK9	HanXRQChr03g0087071	Ha3	-	148424902	148425825	308	1	0	M	6.75	34332.34
HaMKK4	HanXRQChr04g0094171	Ha4	+	471743	472816	351	1	0	C	9.04	38917.18
HaMKK6-1	HanXRQChr09g0238861	Ha9	+	9311933	9322916	357	8	7	-	6.76	39934.36
HaMKK5	HanXRQChr10g0311571	Ha10	+	219604899	219606004	355	1	0	C	9.25	39840.46
HaMKK6-2	HanXRQChr10g0318871	Ha10	+	244056044	244064185	355	8	7	-	7.13	39751.09
HaMKK2	HanXRQChr10g0319531	Ha10	-	245318274	245324118	371	9	8	-	5.43	40967.01
HaMKK1	HanXRQChr12g0354521	Ha12	-	1236278	1243005	358	10	9	-	5.77	39199.81
HaMKK3	HanXRQChr14g0450561	Ha14	-	141579116	141587170	520	12	11	M	5.79	68568.6

**Table 3:** Consensus common docking sites in the MPK proteins belonging to clades A–D.

Clades	Consensus Common Docking Sites
Clade A	K-M-L-V-F-D-P-N-K-R-I-V-E-E-A-L
Clade B	K-M-L-V-F-D-P-S-K-R-I-S-V-T-E-A-L
Clade C	S-L-C-S-W-D-P-C-K-R-P-T-A-E-E-A-L
Clade D	R-L-L-A-F-D-P-K-D-R-P-T-A-E-E-A-L

## Supplementary Materials

Supplementary files are available at http://www.mdpi.com/2223-7747/8/2/28/s1, Table S1: MPKs identified in different species in the present study. Table S2: MKKs identified in different species in the present study. Table S3: Abundance of the MPK gene family of sunflower and ten other species. Table S4: Sunflower MPKs with their orthologs in other plant species. Table S5: Abundance of the MKK gene family of sunflower and ten other species. Table S6: Sunflower MKKs with their orthologs in other plant species. Supplementary Table S7: *k*-means clustering of HaMPK and HaMKK genes. Table S8: miRNAs involved in targeting MPKs and MKKs of Sunflower. Table S9: Tajima’s relative rate test of MPKs. Table S10: Tajima’s relative rate test of MKKs. Table S11: Tajima’s Neutrality Test of MPKs and MKKs. Supplementary Figure S1: Illustrating a MAP Kinase signaling pathway in response to abiotic and biotic stresses in plants adapted from various studies. Supplementary Figure S2: Exon/intron architecture of HaMPK genes. The blue boxes and black lines indicate the exons and introns, respectively. Supplementary Figure S3: Exon/intron architecture of HaMKK genes. The blue boxes and black lines indicate the exons and introns, respectively. Supplementary Figure S4: Maximum Likelihood analysis of full-length MPK amino acid sequences from *A. thaliana* (At), *G. max* (Gm) and *H. annuus* (Ha) using MEGA7 program with 100 bootstrap replicates. *Homo sapiens*, HsMAPK1 (GenBank: NP_002736.3) was used as an outgroup. MPK clades are labeled as A, B, C and D. Supplementary Figure S5: ML tree of full-length MKK amino acid sequences from *A. thaliana* (At), *G. max* (Gm) and *H. annuus* (Ha) in the program MEGA7, with 100 bootstrap replicates. *Homo sapiens*, HsMAPKK1 [GenBank: AAI37460.1] was used as an outgroup. MKK clades are labeled as A, B, C, and D. Supplementary Figure S6: Divergence time estimation of MKK3 sequences from all species used in this study. Supplementary Figure S7: Clustering of sunflower MPK and MKK genes in response to diverse conditions as simulated by salicylic acid (SA), salt (NaCl) and polyethylene glycol (Peg) in leaves and roots. *k*-means clustering method was employed for clustering of genes. Supplementary Figure S7: The log_2_FC values for each HaMPK and HaMKK genes in samples treated with salicylic acid (SA), salt (NaCl) and polyethylene glycol (Peg) in leaves and roots Supplementary File S1: Codes used for RNA-seq data processing. Supplementary File S2: Sequences of the identified MPKs and MKKs including the reference sequences used in this study. Supplementary File S3: MEME predicted conserved motifs in MPK proteins belonging to different phylogenetic clades. Supplementary File S4: MEME predicted conserved motifs in MKK proteins belonging to different phylogenetic clades. Supplementary File S5: Estimated transcript reads for each treatment and their log_2_ fold change values.
