# Supplementary material for: Identification and Characterization of Mitogen-Activated Protein Kinase (MAPK) Genes in Sunflower (Helianthus annuus L.)
Source: Plants (Basel). 2019 Jan 22;8(2):28. doi: 10.3390/plants8020028 (PMC6409774; doi:10.3390/plants8020028)
Supplement: Supplementary file 1 [file plants-08-00028-s001.zip › Supplementary/Table S3, S5.docx]

**Table S3.** The abundance of the MPK gene family of sunflower and ten other species belonging to different clades A-D.

| Gene family | Species | Clade A | Clade B | Clade C | Clade D |
| --- | --- | --- | --- | --- | --- |
| MPK | *Amborella trichopoda* | 5 | 1 | 0 | 2 |
|  | *Aquilegia coerulea* | 5 | 3 | 0 | 3 |
|  | *Arabidopsis thaliana* | 8 | 4 | 0 | 8 |
|  | *Chlamydomonas reinhardtii* | 1 | 2 | 1 | 2 |
|  | *Daucus carota* | 6 | 3 | 0 | 8 |
|  | *Glycine max* | 14 | 4 | 6 | 14 |
|  | *Helianthus annuus* | 9 | 4 | 5 | 10 |
|  | *Oryza sativa* | 3 | 2 | 0 | 10 |
|  | *Solanum lycopersicum* | 4 | 2 | 1 | 8 |
|  | *Sphagnum fallax* | 4 | 2 | 3 | 2 |
|  | *Vitis vinifera* | 5 | 2 | 2 | 5 |

**Table S5.** The abundance of the MKK gene family of sunflower and ten other species belonging to different clades A-D.

| **Gene family** | **Species** | **Clade A** | **Clade B** | **Clade C** | **Clade D** |
| --- | --- | --- | --- | --- | --- |
| MKK | *Amborella trichopoda* | 2 | 1 | 2 | 2 |
|  | *Aquilegia coerulea* | 2 | 1 | 0 | 3 |
|  | *Arabidopsis thaliana* | 3 | 1 | 2 | 4 |
|  | *Chlamydomonas reinhardtii* | 0 | 1 | 0 | 0 |
|  | *Daucus carota* | 2 | 2 | 1 | 1 |
|  | *Glycine max* | 5 | 2 | 2 | 2 |
|  | *Helianthus annuus* | 4 | 1 | 2 | 1 |
|  | *Oryza sativa* | 2 | 1 | 2 | 3 |
|  | *Solanum lycopersicum* | 1 | 1 | 1 | 1 |
|  | *Sphagnum fallax* | 2 | 1 | 0 | 3 |
|  | *Vitis vinifera* | 2 | 1 | 1 | 1 |
