# Supplementary material for: Identification and Characterization of Mitogen-Activated Protein Kinase (MAPK) Genes in Sunflower (Helianthus annuus L.)
Source: Plants (Basel). 2019 Jan 22;8(2):28. doi: 10.3390/plants8020028 (PMC6409774; doi:10.3390/plants8020028)
Supplement: Supplementary file 1 [file plants-08-00028-s001.zip › Supplementary/Table S9, S10, and S11.docx]

# Table S9: Tajima’s relative rate test of MPKs

|  | OsMAPK4, HaMPK6-1, AmtMPK13-1 | OsMAPK4, HaMPK6-1, SfMPK4-1 | OsMAPK4, HaMPK16-1, CreMPK2 |
| --- | --- | --- | --- |
| Identical sites in all three sequences | 213 | 219 | 134 |
| Divergent sites in all three sequences | 22 | 46 | 78 |
| Unique differences in Sequence A | 48 | 25 | 31 |
| Unique differences in Sequence B | 26 | 49 | 64 |
| Unique differences in Sequence C | 28 | 33 | 20 |
| χ2 test | 6.54 | 7.78 | 11.46 |
| P-value | 0.01 | 0.0053 | 0.0007 |
| Degree of freedom | 1 | 1 | 1 |
| Total amino acid positions | 337 | 372 | 327 |

Tajima’s relative rate test to test the equality of evolutionary rate between sequences in column 1: A (OsMAPK4; monocot) and B (HaMPK6, dicot), with sequence C (AmtMPK13-1; basal angiosperm); column 2: A (OsMAPK4; monocot) and B (HaMPK6, dicot), with sequence C (SfMPK4-1; bryophyte); column 3: A (OsMAPK4; monocot) and B (HaMPK16-1, dicot), with sequence C (CreMPK2; algae).

# Table S10. Tajima’s relative rate test of MKKs

| Configuration | OsMAPKK5, HaMKK6-1, AmtMKK6 | OsMKK5, HaMKK6-1, SfMKK3 | OsMKK5, HaMKK6-1, CreMKK3 |
| --- | --- | --- | --- |
| Identical sites in all three sequences | 93 | 81 | 73 |
| Divergent sites in all three sequences | 39 | 125 | 113 |
| Unique differences in Sequence A | 112 | 46 | 41 |
| Unique differences in Sequence B | 4 | 29 | 26 |
| Unique differences in Sequence C | 6 | 43 | 47 |
| χ2 test | 100.55 | 3.85 | 3.36 |
| P-value | 0 | 0.04965 | 0.05687 |
| Degree of freedom | 1 | 1 | 1 |
| Total amino acid positions | 254 | 324 | 300 |

Tajima’s relative rate test to test the equality of evolutionary rate between sequences in column 1: A (OsMAPKK5; monocot) and B (HaMKK6-1, dicot), with sequence C (AmtMKK6; basal angiosperm); column 2: A (OsMAPKK5; monocot) and B (HaMKK6-1, dicot), with sequence C (SfMKK3; bryophyte); column 3: A (OsMAPKK5; monocot) and B (HaMKK6-1, dicot), with sequence C (CreMKK3; algae).

**Table S11**. Tajima’s test for neutrality of MPKs and MKKs

|  | ***m*** | ***S*** | ***p*s** | ***Θ*** | ***π*** | ***D*** | **Total positions** |
| --- | --- | --- | --- | --- | --- | --- | --- |
| **MPK** | 184 | 196 | 0.882883 | 0.152499 | 0.410828 | 5.391062 | 222 |
| **MKK** | 72 | 198 | 0.891892 | 0.184012 | 0.498962 | 5.928839 | 222 |

The analysis involved 184 and 72 amino acid sequences of MPKs and MKKs, respectively. All positions with less than 95% site coverage were eliminated. That is, fewer than 5% alignment gaps, missing data, and ambiguous bases were allowed at any position. There were a total of 222 positions in both MPK and MKK in the final dataset. Evolutionary analyses were conducted in MEGA7. Abbreviations: m = number of sequences, n = total number of sites, S = Number of segregating sites, ps = S/n, Θ = ps/a1, π = nucleotide diversity, and D is the Tajima test statistic.
