# Supplementary material for: Identification and Characterization of Mitogen-Activated Protein Kinase (MAPK) Genes in Sunflower (Helianthus annuus L.)
Source: Plants (Basel). 2019 Jan 22;8(2):28. doi: 10.3390/plants8020028 (PMC6409774; doi:10.3390/plants8020028)
Supplement: Supplementary file 1 [file plants-08-00028-s001.zip › Supplementary/Figure S1.docx]

**Identification and Characterization of Mitogen-Activated Protein Kinase (MAPK) Genes in Sunflower (*Helianthus annuus* L.)**

**Surendra Neupane ^1^, Sarah E. Schweitzer ^1^, Achal Neupane ^1^, Ethan J. Andersen ^1^, Anne Fennell^2^, Ruanbao Zhou^1^, and Madhav P. Nepal^1^***

^1^Department of Biology and Microbiology, South Dakota State University, Brookings, SD 57007, USA

^2^Department of Agronomy, Horticulture and Plant Science, South Dakota State University, Brookings, SD 57007, USA


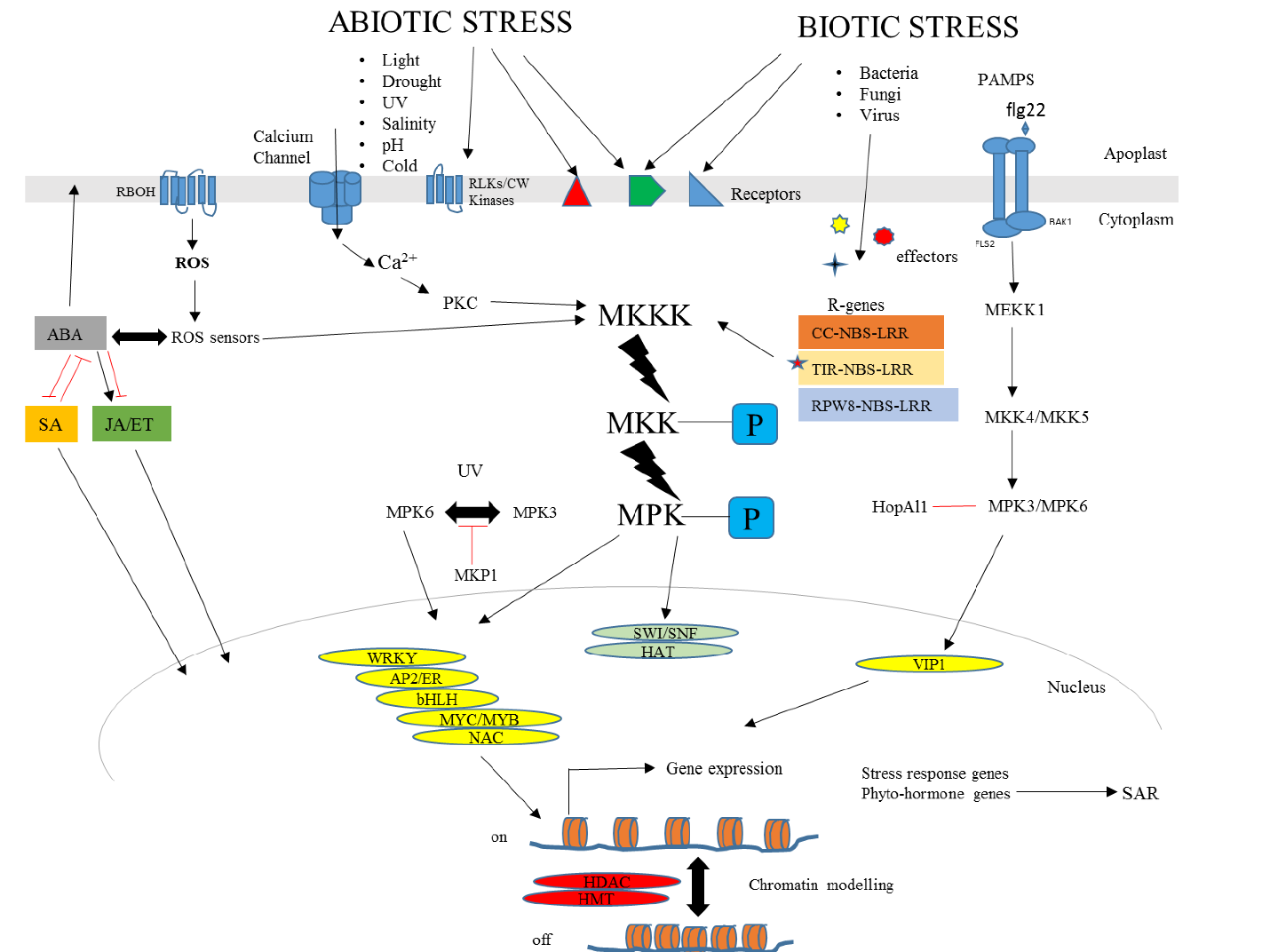


**Figure 1.** Illustrating a MAP Kinase signaling pathway in response to abiotic and biotic stresses in plants adapted from various studies [16, 17, 18, 19, 20, 21, 22, 23, 24]. Diverse abiotic stresses and biotic stresses can potentially change the chemical homeostasis releasing various chemical signals such as Ca^2+^, reactive oxygen species (ROS) in the apoplast of the plant cell [17]. Such signals enter the cytosol via various ways: Ca^2+^ ions enter into the cytosol through calcium channels whereas, ROS such as H_2_O_2_, NO_2_, and O˙_2_ are sensed by respiratory burst oxidase homolog (RBOH) signaling node [16]. Other signaling nodes can be receptor like kinase (RLPs) and cell wall (CW) kinases. In terms of biotic stress, various pathogen associated molecular patterns (PAMPs) are sensed through various receptors. For example, flg22 effectors are sensed by the receptor FLS2/BAK1 [18]. Some pathogens deliver the effector molecules directly into the cell, which are recognized by resistance (R) genes namely, Toll-interleukin-1 receptor-like Nucleotide-binding site Leucine-rich repeat (TNL), Coiled Coil (CC)-NBS-LRR (CNL), and Resistance to powdery mildew8 (RPW8)-NBS-LRR (RNL) genes [22, 23]. All the signals from ROS (via ROS sensors), Ca^2+^ (via calcium dependent protein kinases (PKC), and R genes are mediated through the MAP Kinase signaling pathway. The series of three-tier MAP Kinase phosphorylation events eventually phosphorylate various target regions of transcription factors (WRKY, AP2/ER, bHLH, MYC/MYB, NAC), and actively open the chromatin with the involvement of co-activators such as SWI/SNF and acetylated histones (HAT) [20] that turn on the transcription of stress responsive phytohormone genes [24] [for Systemic Acquired Resistance (SAR)]. On the presence of histone methylase (HMT) and deacetylase (HDAC), the transcription is turned off [20]. The MEKK1→MKK4/MKK5→MPK3/MPK6→VIP1 (virE2 interacting protein 1) cascade is activated when flg22 of the bacterial flagellin interact with the FLS2 receptor during *Pseudomonas syringae* infection [18]. HopAI1 of *P.* *syringae* (phosphothreonine lyase) interact with MPK3 and MPK6 and deactivates downstream signaling [3]. Also, MPK3 and MPK6 are activated by the UV stress in which MAP kinase phosphatase 1 (MKP1) deactivates the signaling [19]. Additionally, Abscisic acid (ABA) plays a major role in adapting abiotic stress [negative effect on Salicylic acid (SA) hormone, positive effect on Jasmonic acid (JA)] signaling and causes callose deposition [16].

References:

16. Kissoudis, C.; van de Wiel, C.; Visser, R.G.; van der Linden, G. Enhancing crop resilience to combined abiotic and biotic stress through the dissection of physiological and molecular crosstalk. *Front. Plant Sci.* **2014**, *5*, 207.

17. Ranty, B.; Aldon, D.; Cotelle, V.; Galaud, J.-P.; Thuleau, P.; Mazars, C. Calcium sensors as key hubs in plant responses to biotic and abiotic stresses. *Front. Plant Sci.* **2016**, *7*, 327.

18. Pitzschke, A.; Schikora, A.; Hirt, H. MAPK cascade signalling networks in plant defence. *Curr. Opin. Plant Biol.* **2009**, *12*, 421-426.

19. Müller-Xing, R.; Xing, Q.; Goodrich, J. Footprints of the sun: memory of UV and light stress in plants. *Front. Plant Sci.* **2014**, *5*, 474.

20. Davis, P.K.; Brachmann, R.K. Chromatin remodeling and cancer. *Cancer Biol. Therapy* **2003**, *2*, 23-30.

21. Rejeb, I.B.; Pastor, V.; Mauch-.Mani, B. Plant responses to simultaneous biotic and abiotic stress: molecular mechanisms. *Plants.* **2014**, *3*, 458-475.

22. Meyers, B.C.; Kozik, A.; Griego, A.; Kuang, H.; Michelmore, R.W. Genome-wide analysis of NBS-LRR-encoding genes in *Arabidopsis*. *Plant Cell.* **2003**, *15*.

23. Shao, Z.-Q.; Xue, J.-Y.; Wu, P.; Zhang, Y.-M.; Wu, Y.; Hang, Y.-Y.; Wang, B.; Chen, J.-Q. Large-scale analyses of angiosperm nucleotide-binding site-leucine-rich repeat (NBS-LRR) genes reveal three anciently diverged classes with distinct evolutionary patterns. *Plant Physiol.* **2016**, pp. 01487.02015.

24. Ramirez-Prado, J.S.; Abulfaraj, A.A.; Rayapuram, N.; Benhamed, M.; Hirt, H. Plant immunity: from signaling to epigenetic control of defense. *Trends Plant Sci.* **2018**, 9, 833-844.
