# Supplementary material for: Identification and Characterization of Mitogen-Activated Protein Kinase (MAPK) Genes in Sunflower (Helianthus annuus L.)
Source: Plants (Basel). 2019 Jan 22;8(2):28. doi: 10.3390/plants8020028 (PMC6409774; doi:10.3390/plants8020028)

Supplementary File S4. MEME predicted conserved motifs in MKK proteins belonging to different phylogenetic clades. A set of parameters used in MEME analysis included maxsize: 100,000, mod: zoops, nmotifs: 10, minw: 6, and maxw: 25.

Clade A

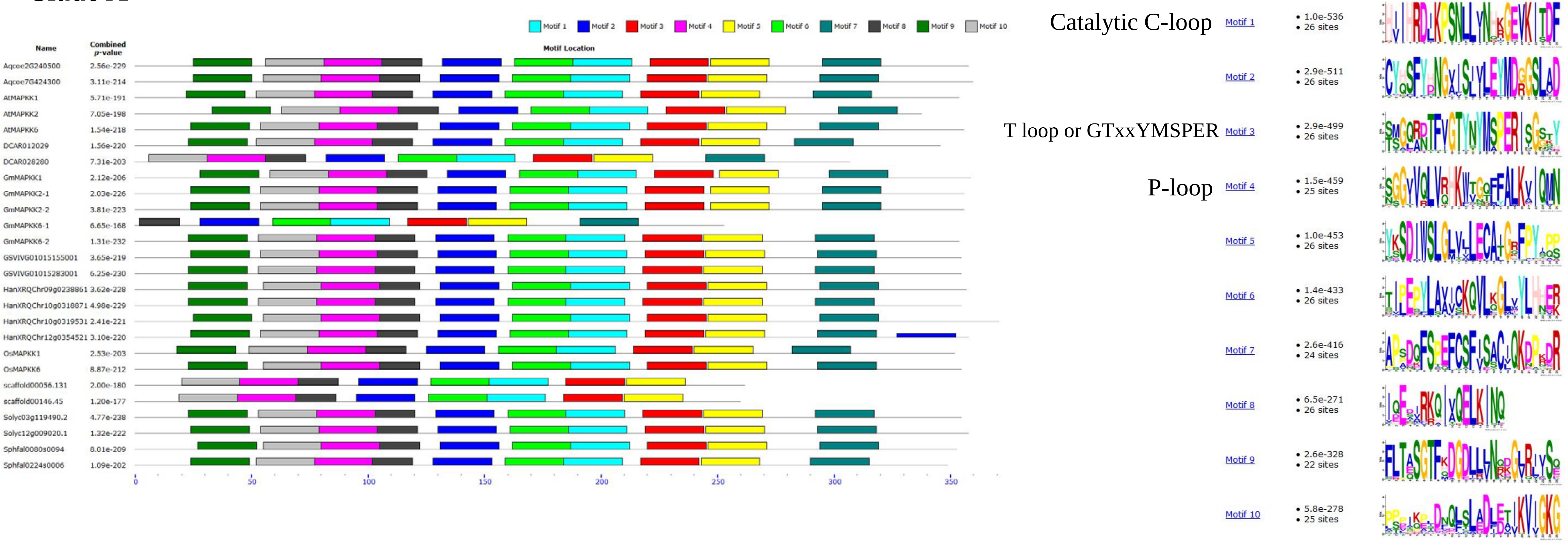

Clade B

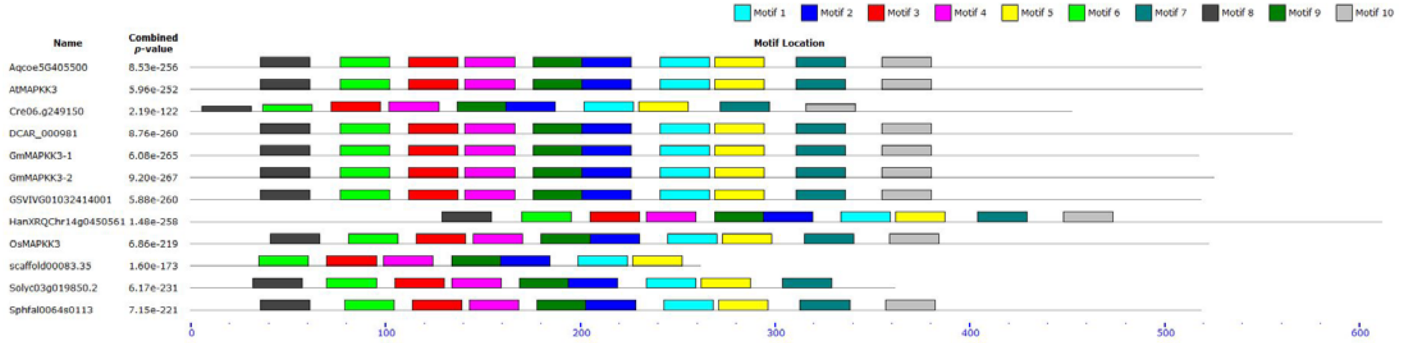

T loop or GTxxYMSPER	Motif 1	1.7e-238	12 sites
Catalytic C-loop	Motif 2	2.1e-211	12 sites
	Motif 3	6.7e-207	12 sites
	Motif 4	5.5e-201	12 sites
	Motif 5	1.6e-187	12 sites
P-loop	Motif 6	6.5e-182	11 sites
	Motif 7	3.2e-158	11 sites
	Motif 8	2.1e-145	10 sites
	Motif 9	5.8e-132	12 sites
	Motif 10	1.0e-125	9 sites

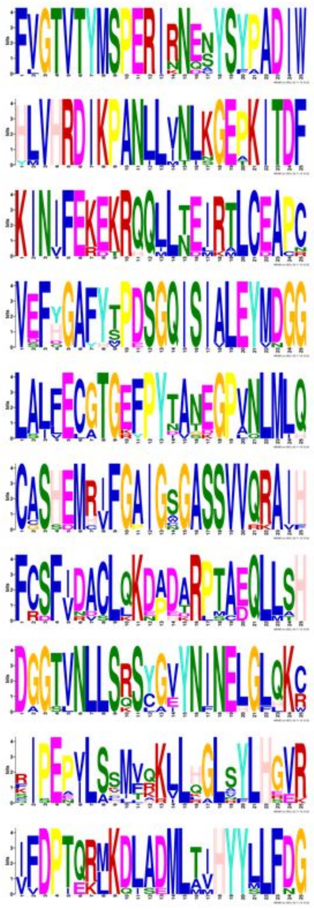

Clade C

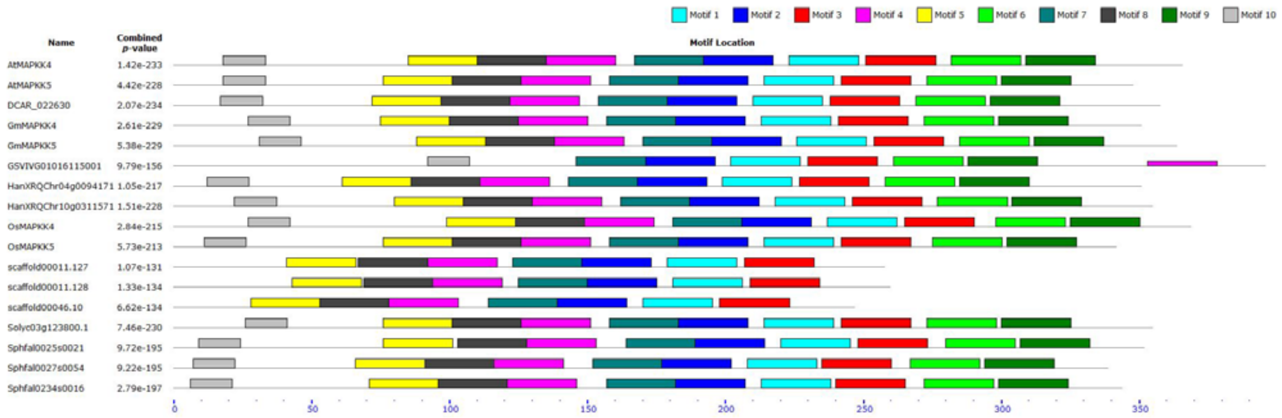

T loop or GTxxYMSPER — Motif 1 — 8.9e-331, 17 sites

Catalytic C-loop — Motif 2 — 1.3e-310, 17 sites

Motif 3 — 3.3e-292, 17 sites

Motif 4 — 7.4e-252, 16 sites

P-loop — Motif 5 — 4.9e-214, 16 sites

Motif 6 — 5.6e-225, 14 sites

Motif 7 — 6.7e-186, 17 sites

Motif 8 — 3.3e-179, 16 sites

Motif 9 — 6.9e-186, 14 sites

Motif 10 — 2.5e-069, 14 sites

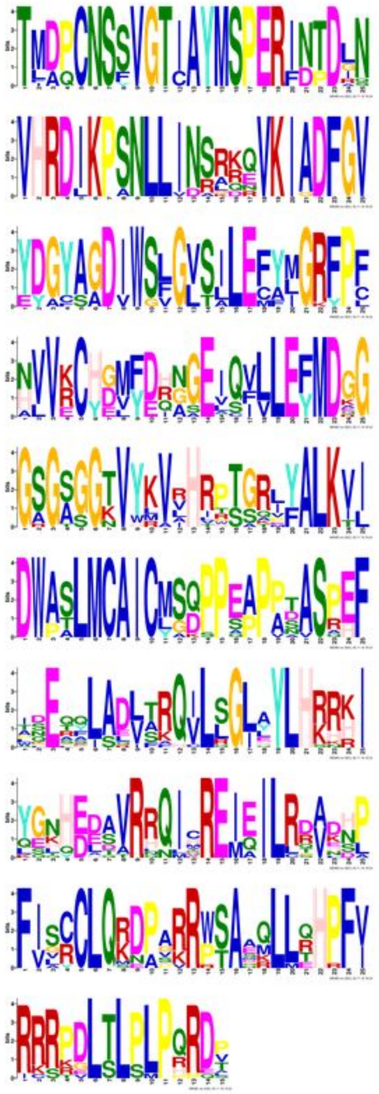

Clade D

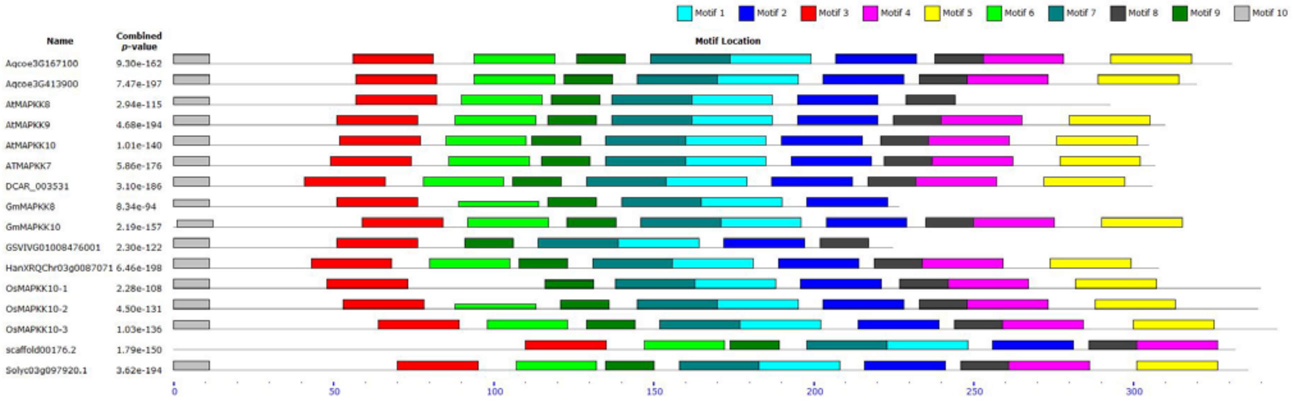

Catalytic C-loop	Motif 1	• 1.0e-243 • 16 sites	
T loop or GTxxYMSPER	Motif 2	• 2.3e-213 • 16 sites	
P-loop	Motif 3	• 5.0e-202 • 16 sites	
	Motif 4	• 5.9e-190 • 13 sites	
	Motif 5	• 1.1e-129 • 12 sites	
	Motif 6	• 1.8e-112 • 12 sites	
	Motif 7	• 1.5e-109 • 16 sites	
	Motif 8	• 4.1e-105 • 15 sites	
	Motif 9	• 7.2e-085 • 16 sites	
	Motif 10	• 1.8e-040 • 15 sites	

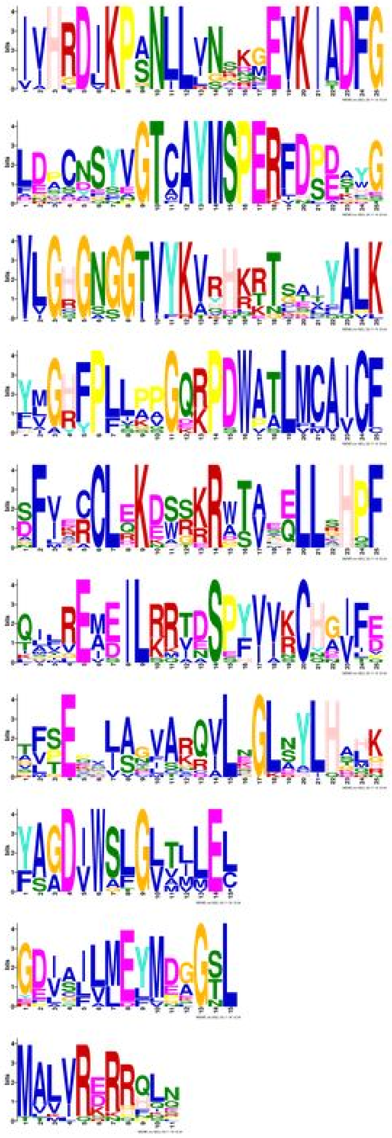

Supplement: Supplementary file 1 [file plants-08-00028-s001.zip › Supplementary/Supplementary File S4.pdf]
