# Supplementary material for: Identification and Characterization of Mitogen-Activated Protein Kinase (MAPK) Genes in Sunflower (Helianthus annuus L.)
Source: Plants (Basel). 2019 Jan 22;8(2):28. doi: 10.3390/plants8020028 (PMC6409774; doi:10.3390/plants8020028)
Supplement: Supplementary file 1 [file plants-08-00028-s001.zip › Supplementary/Supplementary File S3.pdf]

Supplementary File S3. MEME predicted conserved motifs in MPK proteins belonging to different phylogenetic clades. A set of parameters used in MEME analysis included maxsize: 100,000, mod: zoops, nmotifs: 10, minw: 6, and maxw: 25.

Clade A

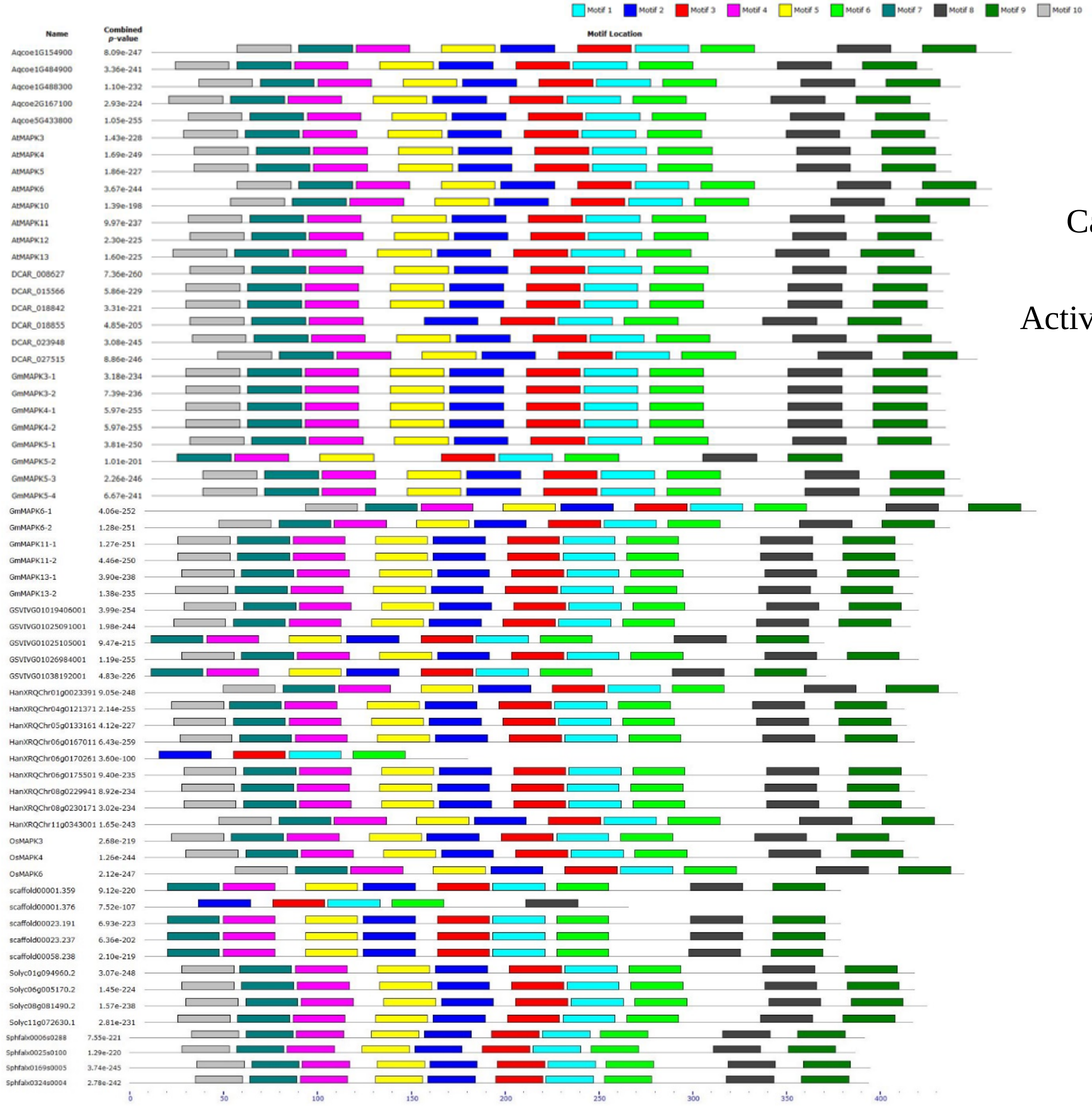

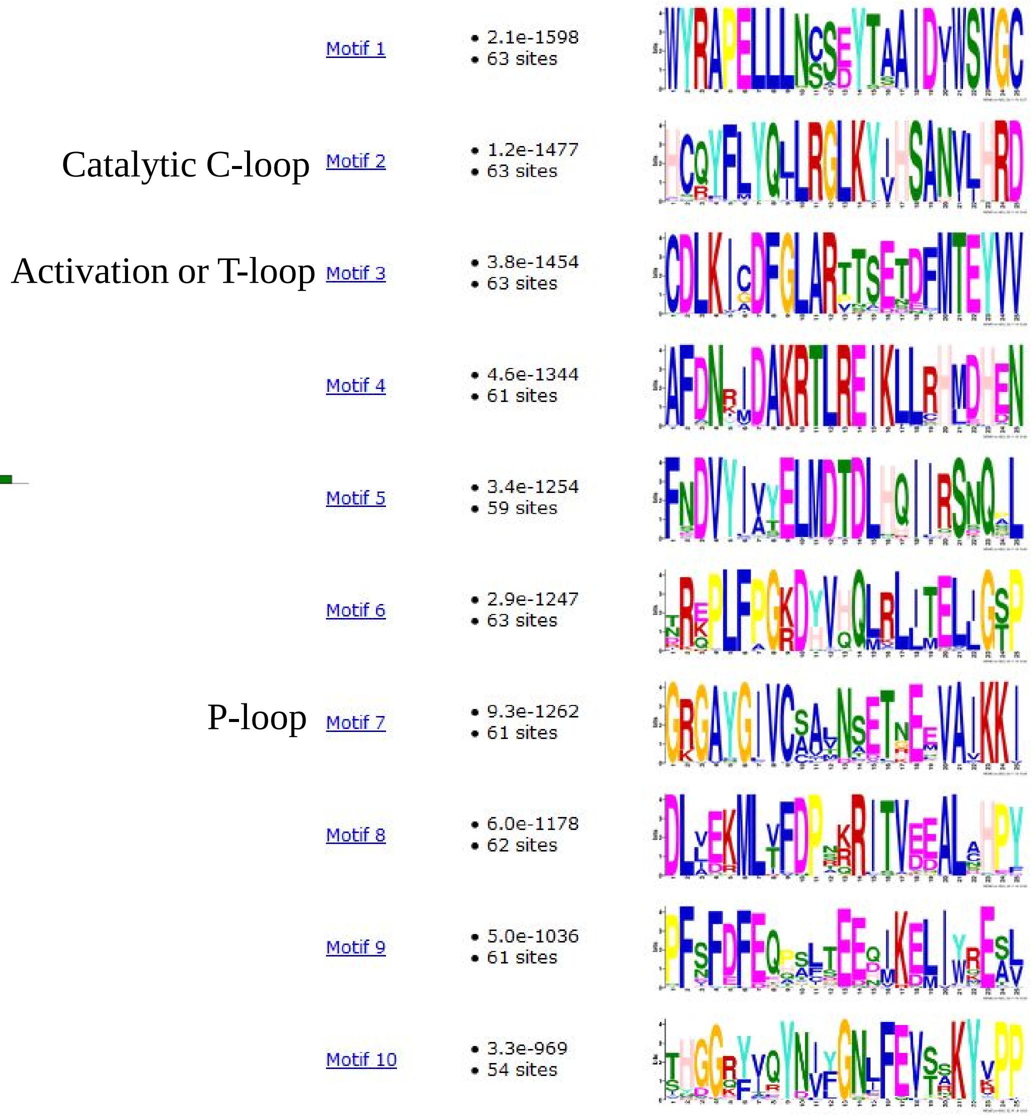

Clade B

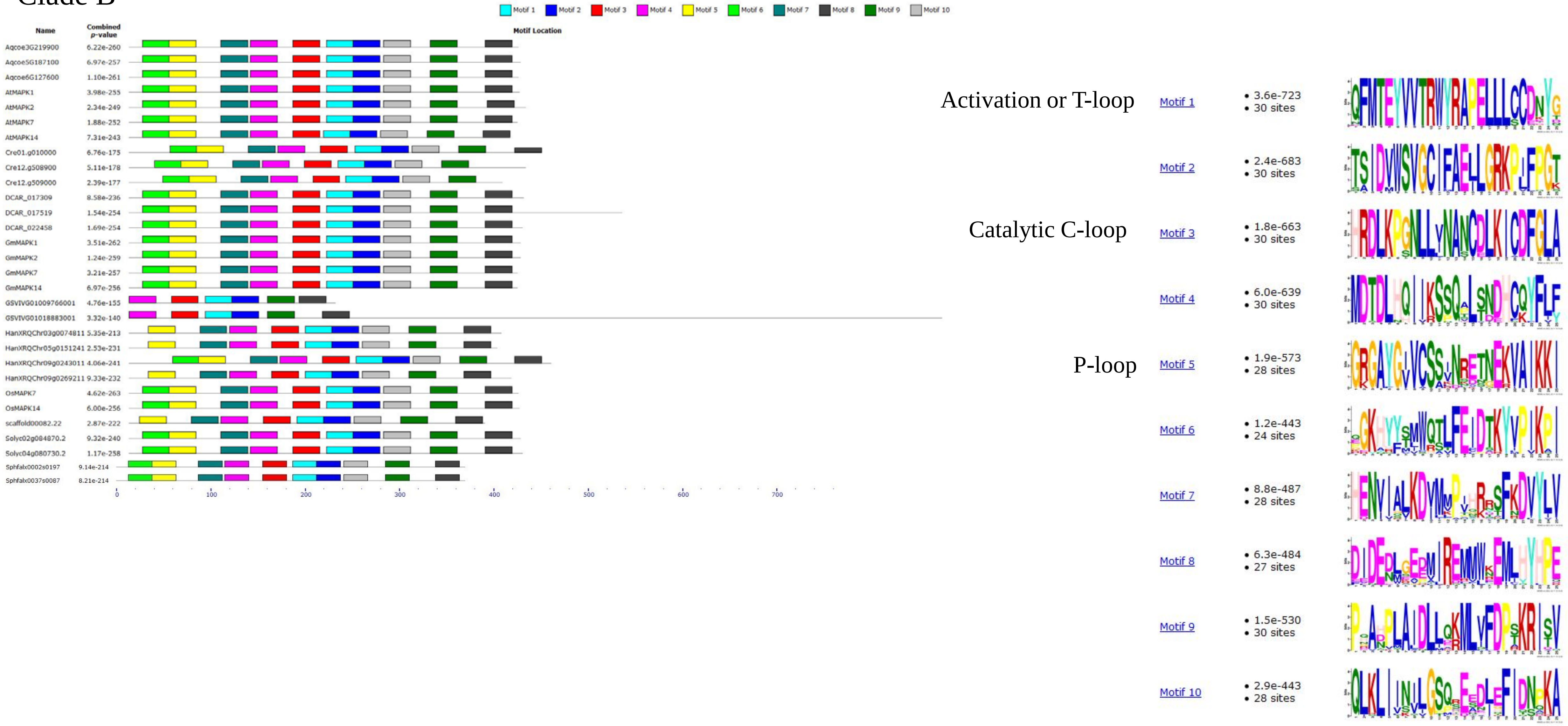

Motif 1
Motif 2
Motif 3
Motif 4
Motif 5
Motif 6
Motif 7
Motif 8
Motif 9
Motif 10
Name
Combined p-value
Motif Location
Aqcoe3G219900 6.22e-260
Aqcoe5G187100 6.97e-257
Aqcoe6G127600 1.10e-261
AtMAPK1 3.98e-255
AtMAPK2 2.34e-249
AtMAPK7 1.88e-252
AtMAPK14 7.31e-243
Cre01.g010000 6.76e-175
Cre12.g508900 5.11e-178
Cre12.g509000 2.39e-177
DCAR_017309 8.58e-236
DCAR_017519 1.54e-254
DCAR_022458 1.69e-254
GmMAPK1 3.51e-262
GmMAPK2 1.24e-259
GmMAPK7 3.21e-257
GmMAPK14 6.97e-256
GSVIVG01009766001 4.76e-155
GSVIVG01018883001 3.32e-140
HanXRQChr03g0074811 5.35e-213
HanXRQChr05g0151241 2.53e-231
HanXRQChr09g0243011 4.06e-241
HanXRQChr09g0269211 9.33e-232
OsMAPK7 4.62e-263
OsMAPK14 6.00e-256
scaffold00082.22 2.87e-222
Solyc02g084870.2 9.32e-240
Solyc04g080730.2 1.17e-258
Sphfalx0002s0197 9.14e-214
Sphfalx0037s0087 8.21e-214
0
100
200
300
400
500
600
700
Activation or T-loop
Motif 1
• 3.6e-723
• 30 sites
Motif 2
• 2.4e-683
• 30 sites
Catalytic C-loop
Motif 3
• 1.8e-663
• 30 sites
Motif 4
• 6.0e-639
• 30 sites
P-loop
Motif 5
• 1.9e-573
• 28 sites
Motif 6
• 1.2e-443
• 24 sites
Motif 7
• 8.8e-487
• 28 sites
Motif 8
• 6.3e-484
• 27 sites
Motif 9
• 1.5e-530
• 30 sites
Motif 10
• 2.9e-443
• 28 sites

Clade C

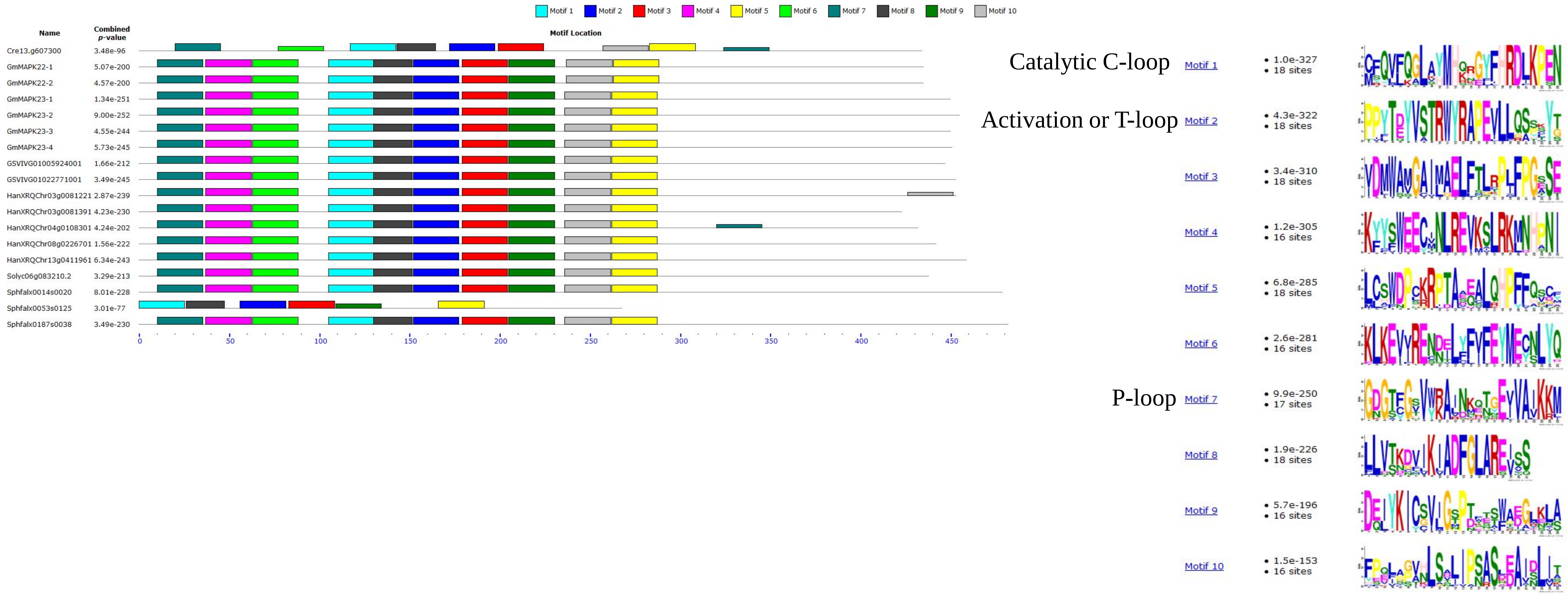

Motif 1 Motif 2 Motif 3 Motif 4 Motif 5 Motif 6 Motif 7 Motif 8 Motif 9 Motif 10
Name
Combined p-value
Motif Location
Cre13.g607300 3.48e-96
GmMAPK22-1 5.07e-200
GmMAPK22-2 4.57e-200
GmMAPK23-1 1.34e-251
GmMAPK23-2 9.00e-252
GmMAPK23-3 4.55e-244
GmMAPK23-4 5.73e-245
GSVIVG01005924001 1.66e-212
GSVIVG01022771001 3.49e-245
HanXRQChr03g0081221 2.87e-239
HanXRQChr03g0081391 4.23e-230
HanXRQChr04g0108301 4.24e-202
HanXRQChr08g0226701 1.56e-222
HanXRQChr13g0411961 6.34e-243
Solyc06g083210.2 3.29e-213
Sphfalx0014s0020 8.01e-228
Sphfalx0053s0125 3.01e-77
Sphfalx0187s0038 3.49e-230
0 50 100 150 200 250 300 350 400 450
Catalytic C-loop
Motif 1 • 1.0e-327 • 18 sites
Activation or T-loop
Motif 2 • 4.3e-322 • 18 sites
Motif 3 • 3.4e-310 • 18 sites
Motif 4 • 1.2e-305 • 16 sites
Motif 5 • 6.8e-285 • 18 sites
Motif 6 • 2.6e-281 • 16 sites
P-loop
Motif 7 • 9.9e-250 • 17 sites
Motif 8 • 1.9e-226 • 18 sites
Motif 9 • 5.7e-196 • 16 sites
Motif 10 • 1.5e-153 • 16 sites

Clade D

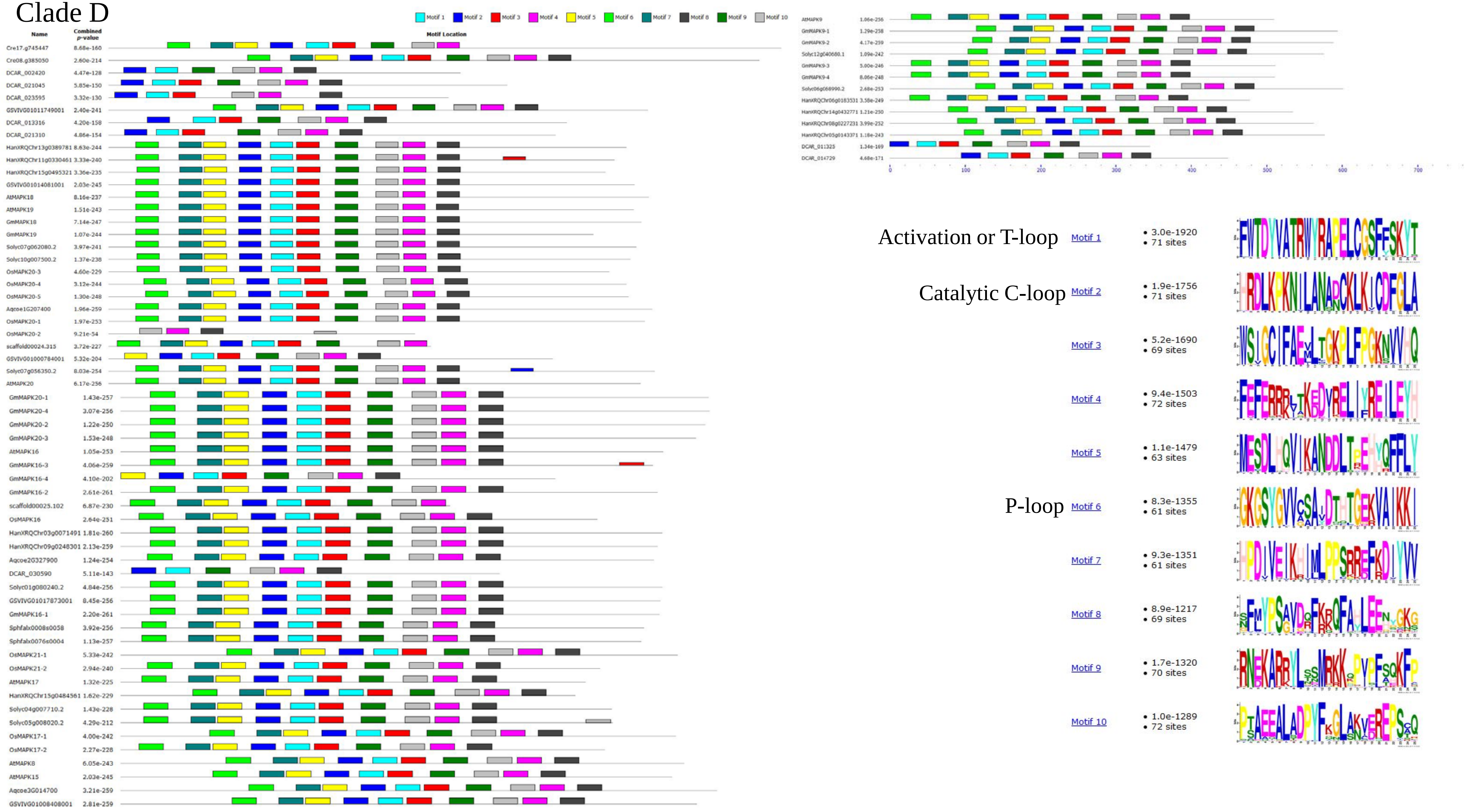

Motif 1
Motif 2
Motif 3
Motif 4
Motif 5
Motif 6
Motif 7
Motif 8
Motif 9
Motif 10
Name
Combined p-value
Motif Location
Activation or T-loop
Motif 1
• 3.0e-1920
• 71 sites
Catalytic C-loop
Motif 2
• 1.9e-1756
• 71 sites
Motif 3
• 5.2e-1690
• 69 sites
Motif 4
• 9.4e-1503
• 72 sites
Motif 5
• 1.1e-1479
• 63 sites
P-loop
Motif 6
• 8.3e-1355
• 61 sites
Motif 7
• 9.3e-1351
• 61 sites
Motif 8
• 8.9e-1217
• 69 sites
Motif 9
• 1.7e-1320
• 70 sites
Motif 10
• 1.0e-1289
• 72 sites
