# Supplementary figures and images for: Identification and Characterization of Mitogen-Activated Protein Kinase (MAPK) Genes in Sunflower (Helianthus annuus L.)
Source: Plants (Basel). 2019 Jan 22;8(2):28. doi: 10.3390/plants8020028 (PMC6409774; doi:10.3390/plants8020028)

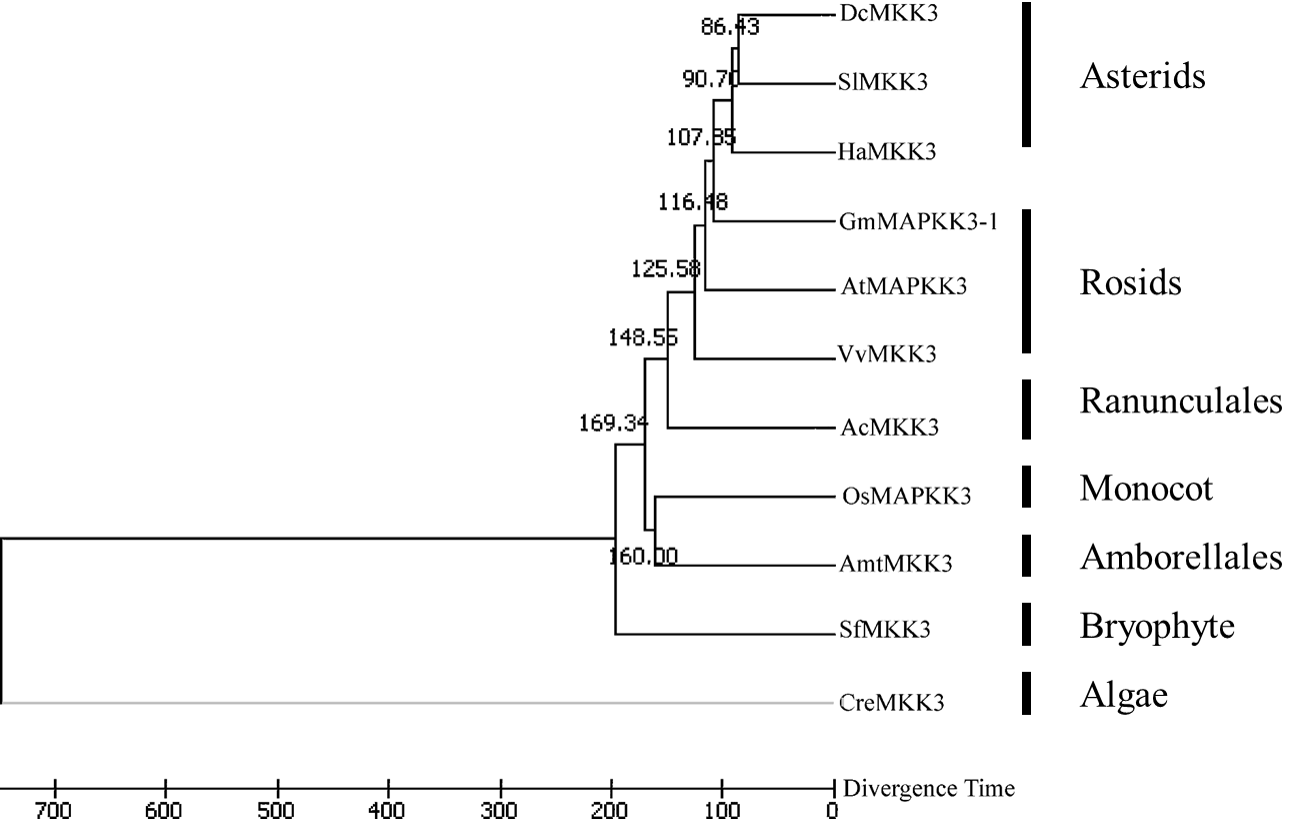

Supplement: Supplementary file 1 [file plants-08-00028-s001.zip › Supplementary/Figure S6.tif]

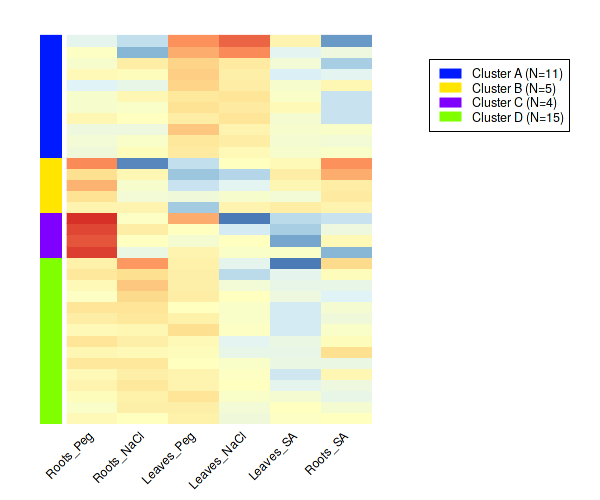

Supplement: Supplementary file 1 [file plants-08-00028-s001.zip › Supplementary/Figure S7.png]

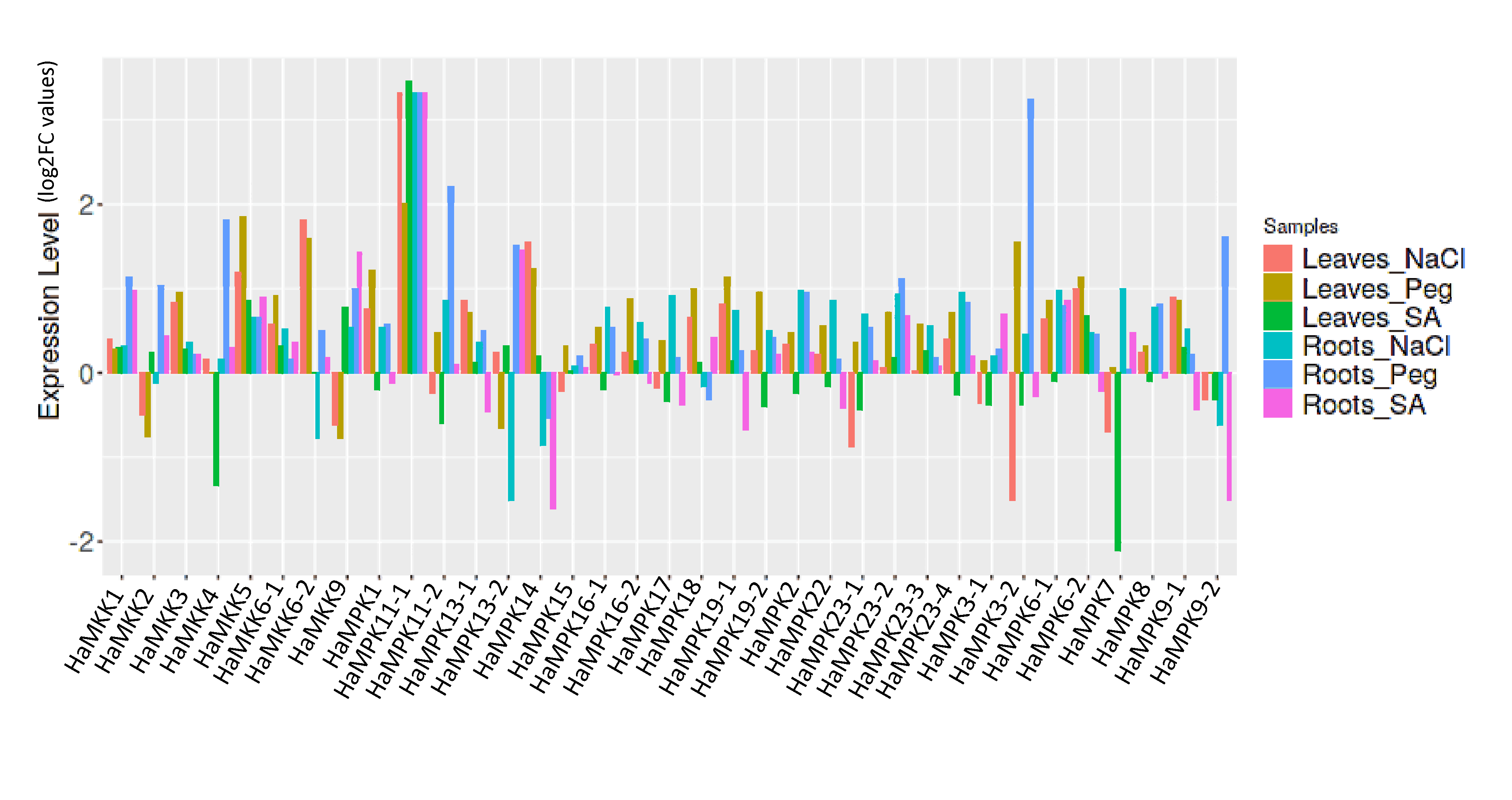

Supplement: Supplementary file 1 [file plants-08-00028-s001.zip › Supplementary/Figure S8.tif]

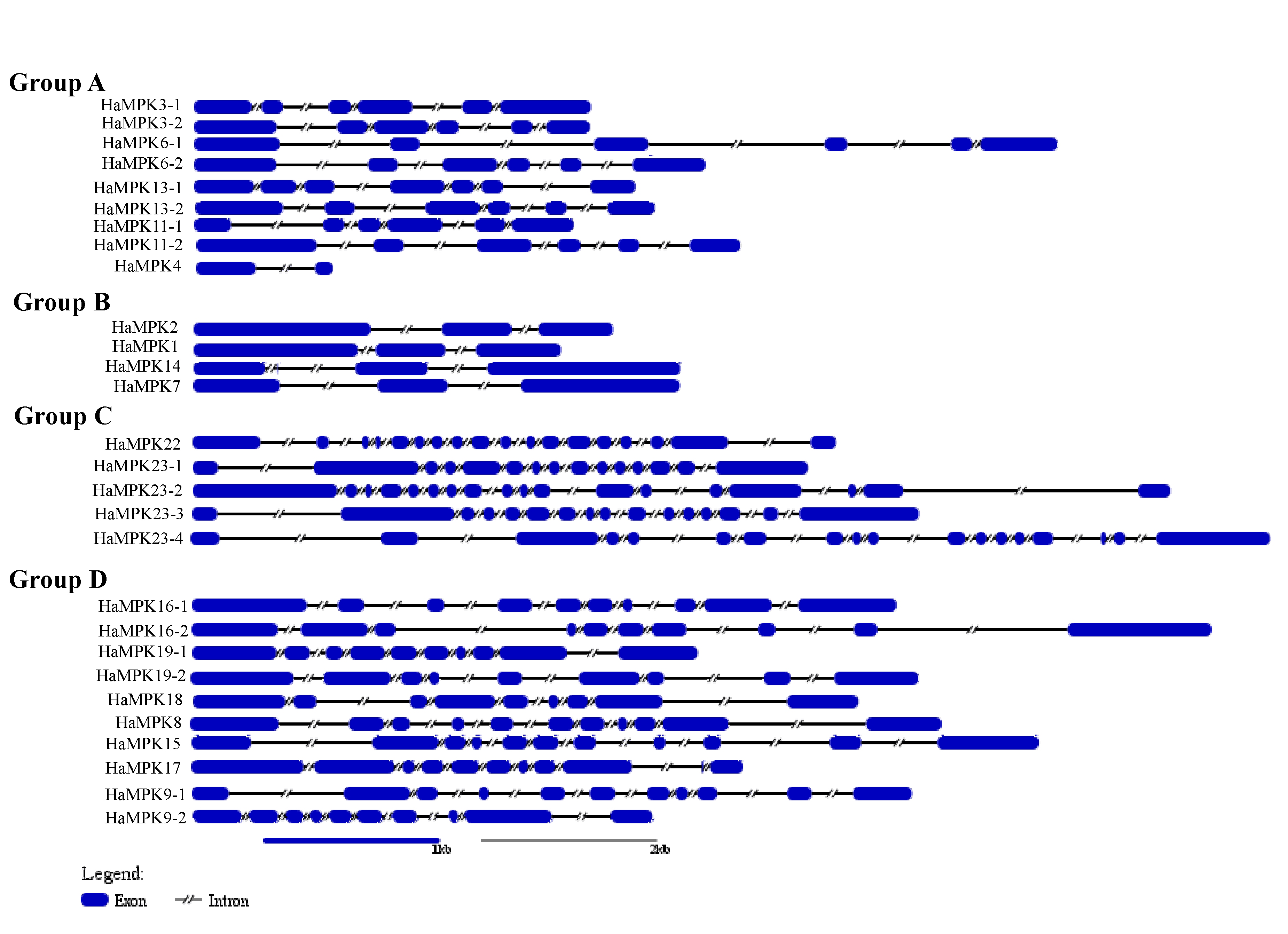

Supplement: Supplementary file 1 [file plants-08-00028-s001.zip › Supplementary/Figure S2.tif]

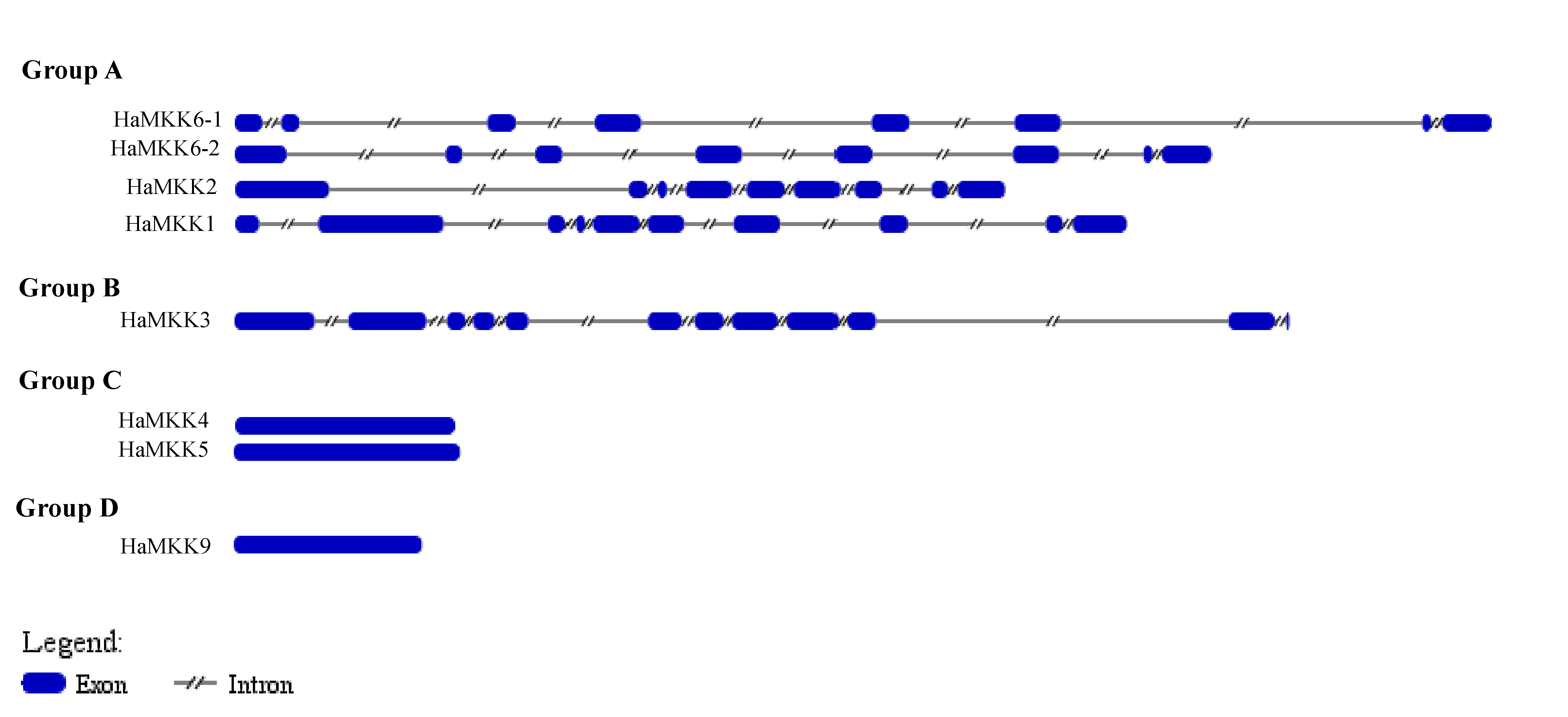

Supplement: Supplementary file 1 [file plants-08-00028-s001.zip › Supplementary/Figure S3.tif]

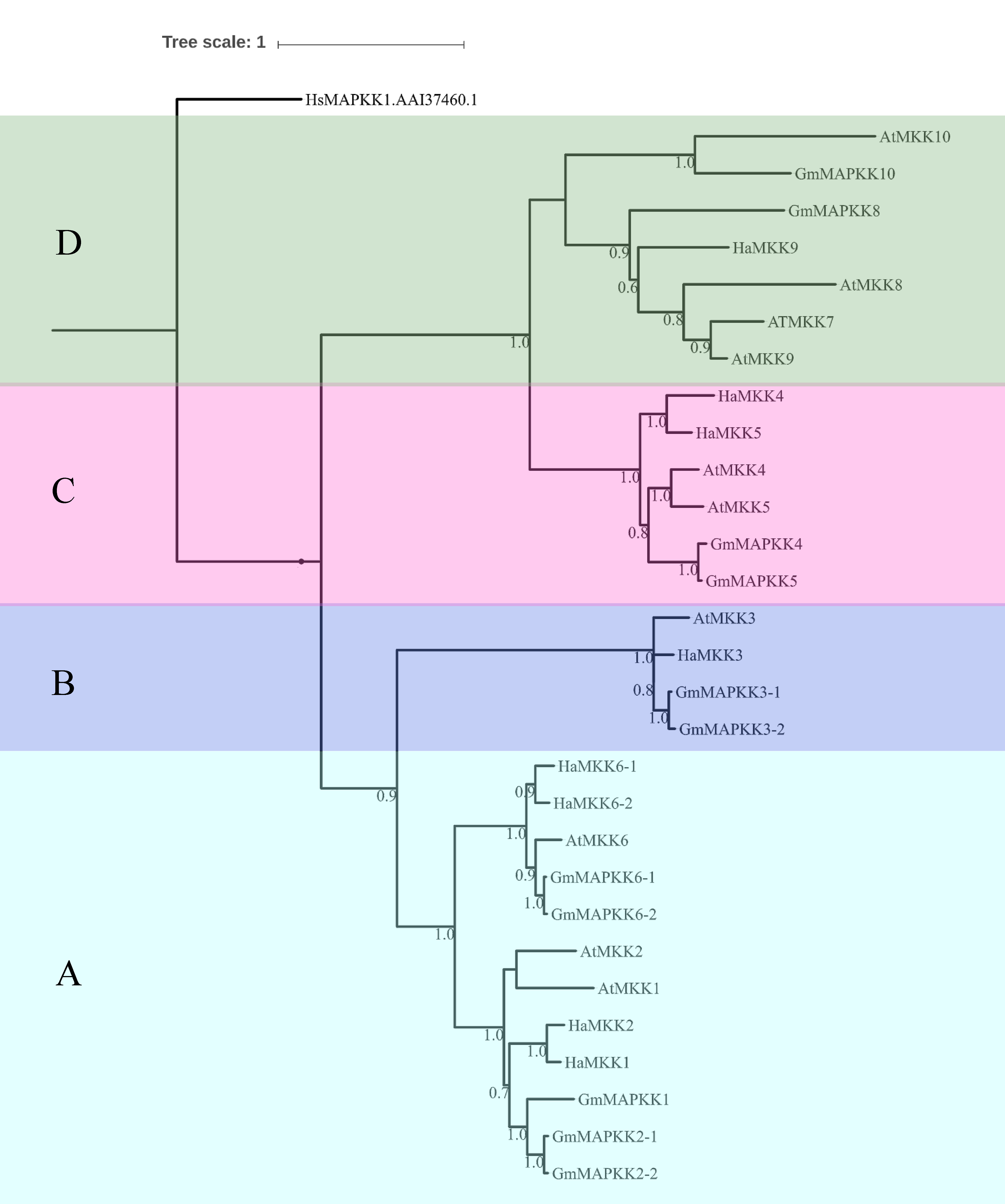

Supplement: Supplementary file 1 [file plants-08-00028-s001.zip › Supplementary/Figure S5.tif]

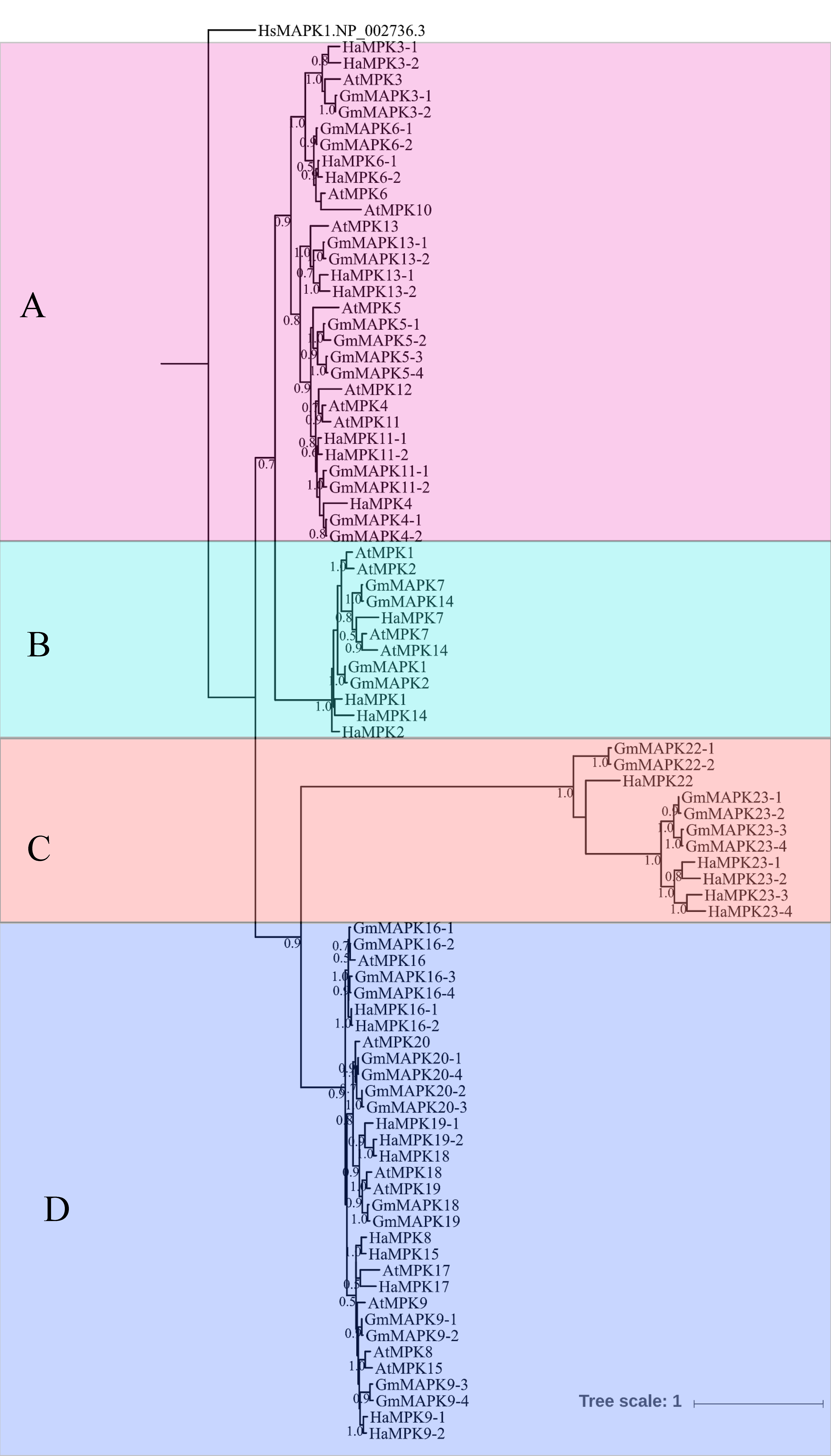

Supplement: Supplementary file 1 [file plants-08-00028-s001.zip › Supplementary/Figure S4.tif]
